# A Golden Fullerene Encapsulating Schmid Gold

**DOI:** 10.1021/jacs.5c20164

**Published:** 2026-01-15

**Authors:** Peiyao Pan, Sami Malola, Rui Zhao, Wentao Huang, Emmi Pohjolainen, María Francisca Matus, Meng Zhou, Xi Kang, Hannu Häkkinen, Manzhou Zhu

**Affiliations:** † Department of Chemistry and Centre for Atomic Engineering of Advanced Materials, Key Laboratory of Structure and Functional Regulation of Hybrid Materials of Ministry of Education, Anhui Province Key Laboratory of Chemistry for inorganic/Organic Hybrid Functionalized Materials, Anhui University, Hefei, Anhui 230601, P. R. China; ‡ Departments of Physics and Chemistry, Nanoscience Center, University of Jyväskylä, Jyväskylä FI-40014, Finland; § Hefei National Research Center for Physical Sciences at the Microscale, Department of Chemical Physics, University of Science and Technology of China, Hefei, Anhui 230026, China; ∥ State Key Laboratory of Optoelectronic Information Acquisition and Protection Technology, Hefei, Anhui 230601, China

## Abstract

Since its first synthesis in 1981, determining the structure of the “Schmid gold”—initially determined as a Au_55_(PPh_3_)_12_Cl_6_ cluster—has remained a big challenge. In this study, fluorine chemistry is exploited to stabilize the largest structurally resolved gold nanocluster stabilized by phosphine ligands, Au_75_(P­(C_6_H_4_-4-CF_3_)_3_)_20_Cl_12_, with which the atomically precise structure of the Schmid gold was optimized. The Au_75_ nanocluster displays a Russian doll-like shell-by-shell configuration of Au_13_@Au_42_@Au_20_@Cl_12_@(PR)_20_. The first two shells resemble the metallic core of the Schmid gold, which is encapsulated by an Au_20_ shell showcasing a fullerene-like topology. Consequently, the structure of the Au_75_ nanocluster is referred to as the “golden fullerene encapsulating Schmid gold”. The geometric constraints of Au_20_@Au_55_ dominate over size effects in dictating photodynamics in the Au_75_ nanocluster. Density functional theory analysis revealed the superatomic character of the fluorinated Au_75_ nanocluster and molecular dynamics simulations up to three microsecond time scale confirmed the role of fluorine chemistry in stabilizing its structure. This study demonstrates the potential to stabilize large phosphine-protected gold nanoclusters by fluorine chemistry opening doors to understanding their functionality in catalysis and biological applications.

## Graphical abstract

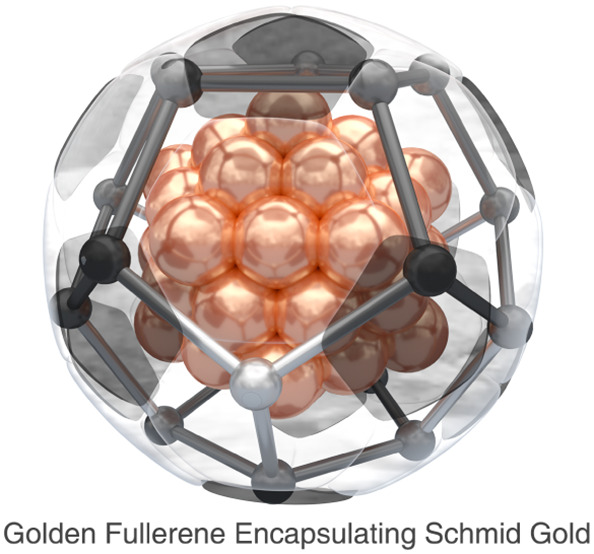

## Introduction

Gold nanoparticles have garnered significant attention due to their unique electronic, optical, and catalytic properties.^1–6^ Since Faraday reported colloidal gold in 1857,^7^ the precise synthesis and atomic-level structural determination of these nanomaterials have remained at the forefront of nanoscience. A key challenge in their development lies in the stability control of ultrasmall-sized (2 nm or smaller) nanoparticles which, historically, has been achieved with either phosphine or thiol chemistry. In 1994, Brust, Schiffrin and collaborators published a recipe^8^ to stabilize small gold nanoparticles with thiols, which has led to numerous variants of the so-called “Brust-Schiffrin” synthesis yielding hundreds of various atomically precise thiolate-protected noble metal clusters.^6,9–28^ In contrast, the roots of gold-phosphine chemistry go back to 1969 when McPartlin, Mason and Malatesta discovered the “undecagold” Au_11_ cluster stabilized with seven phosphine and three electronegative ligands.^29^ Although phosphine chemistry proved initially successful for achieving atom-precise structural characterization of very small gold clusters, the largest crystallographically resolved phosphine-stabilized cluster to date is still Au_39_(PPh_3_)_14_Cl_6_ from 1992 ^30^ while thiol chemistry has yielded very large accurately resolved clusters up to size Au_279_.^22^

Among the phosphine-stabilized gold clusters, the “Schmid gold” with an initial formula of Au_55_(PPh_3_)_12_Cl_6_ has received widespread attention since 1981 due to its unique electronic properties and promising applications in nanoelectronics, catalysis and biology,^31–35^ leading even to commercial bioimaging products. Despite its widespread interest, the atomically precise structure of the “Schmid gold” remains elusive. Significant efforts—such as extended X-ray absorption fine structure, powder X-ray diffraction, high-resolution transmission electron microscopy, X-ray scattering, analytical ultracentrifugation, aberration-corrected scanning transmission electron microscopy, and theoretical calculations—have been made to determine the possible structure of the “Schmid gold”. As a result, various structural forms have been suggested, including cuboctahedron, icosahedron, hybrid icosahedron-cuboctahedron, and even amorphous structure.^36–43^

It has been acknowledged that the relatively weak phosphine-gold bond is the culprit for the failure to grow large enough, good quality crystals for definite structural determinations of the “Schmid gold”.^34^ Recently, fluorochemical modification has been exploited to address the crystallization challenges in several research fields.^44–46^ The fluorine chemistry improves the crystallizability of nanomaterials from two key aspects: (i) enhancing structural rigidity through the replacement of hydrogen with fluorine at the molecule level; (ii) promoting long-range ordered arrangements of molecules through supramolecular fluorine–fluorine interactions. In this context, we perceived a promising opportunity to stabilize and crystallize metal nanoclusters with inaccessible structures.

Here, with fluorination of triphenylphosphine ligands, we report the largest structurally resolved phosphine-protected gold nanocluster to date, Au_75_(P­(C_6_H_4_-4-CF_3_)_3_)_20_Cl_12_. The Au_75_ nanocluster follows a Russian doll-like shell-by-shell configuration of Au_13_@Au_42_@Au_20_@Cl_12_@(PR)_20_, wherein the first and second shells enlighten the metallic kernel of the “Schmid gold” and the third shell showcases a fullerene-like topology, similar to the smallest fullerene C_20_. Strong F-initiated interactions are observed at both molecule and supramolecular levels, endowing the Au_75_ nanocluster with strong stability and good crystallinity. Transient absorption analyses indicate that Au_75_ exhibits unique excited-state dynamics that transcend conventional size classifications. Density functional theory calculations shed light onto the electronic structure of this novel cluster as well as onto the stabilizing role of the C_20_-symmetric [Au­(P­(C_6_H_4_-4-CF_3_)_3_)]_20_ mantle around the Au_55_ kernel, which is also demonstrated via extensive molecular dynamics simulations of the cluster in solvent using a dedicated classical force field developed in this work. While shedding light to the long-standing controversy of “Schmid gold”, our work opens doors for synthetic control and characterization of large phosphine-stabilized gold nanoclusters via fluorination chemistry (Figure 1).

**Figure 1:**
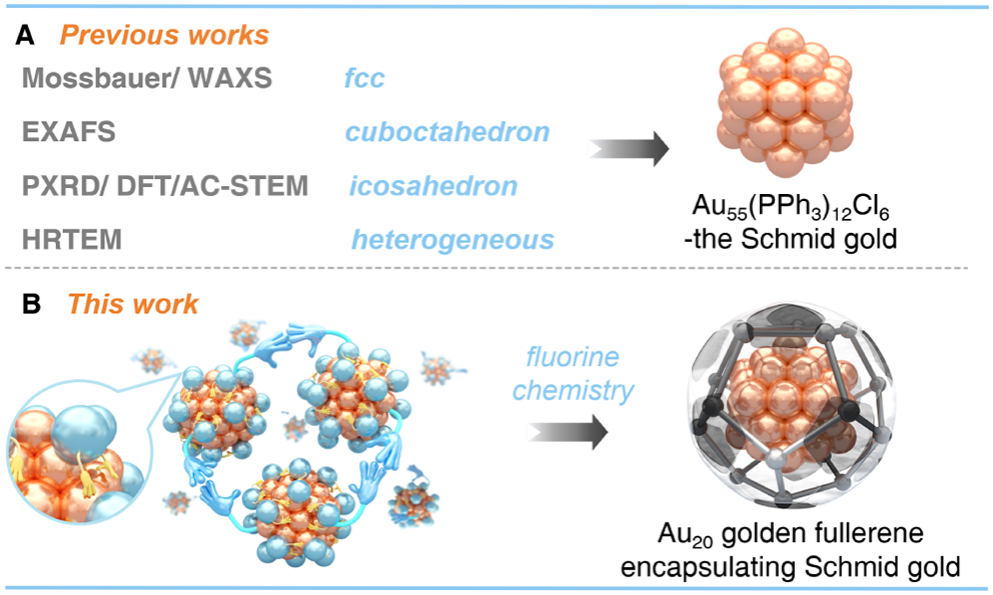
Previous efforts (A) and this work (B) in determining the structure of “Schmid gold”.

## Results and Discussion

The Au_75_(P­(C_6_H_4_-4-CF_3_)_3_)_20_Cl_12_ nanocluster, featuring fluorinated phosphine ligands, is synthesized using a one-pot method. The process involved the sequential addition of HAuCl_4_, P­(C_6_H_4_-4-CF_3_)_3_, and cyclohexyl mercaptan to a mixed solvent of methanol and dichloromethane. After stirring for 30 min, a freshly prepared aqueous solution of NaBH_4_ was added dropwise. The reaction continued for 10 h, after which centrifugation was employed to remove insoluble impurities. The resulting supernatant was evaporated to dryness, yielding a green solid that was then washed with *n*-hexane, resulting in the preparation of the Au_75_ nanocluster. High-quality crystals of the Au_75_ nanocluster were obtained by diffusing *n*-hexane into the dichloromethane solution containing the nanocluster over 1 week (Figure S1). The cyclohexyl mercaptan is crucial for the formation of the Au_75_ nanocluster; its absence results in the generation of an undesired product, Au_11_(P­(C_6_H_4_-4-CF_3_)_3_)_7_Cl_3_ (Figure S2). In addition, the presence of fluorinated phosphine ligands is essential for synthesizing Au_75_(P­(C_6_H_4_-4-CF_3_)_3_)_20_Cl_12_, as nonfluorinated PPh_3_ ligands lead to the formation of the smaller Au_25_(PPh_3_)_10_(SC_6_H_11_)_5_Cl_2_ nanocluster (Figure S2). In this context, the involvement of fluorine chemistry is beneficial for the successful synthesis of the Au_75_ nanocluster, which, to the best of our knowledge, is the largest structurally resolved gold nanocluster stabilized by phosphine ligands (Figure S3).

X-ray photoelectron spectroscopy identifies an Au 4f_7/2_ binding energy of 83.8 eV, indicating that the gold atoms in the Au_75_ nanocluster primarily exist in the Au^0^ oxidation state (Figure S4). Thermogravimetric analysis reveals an experimental weight loss of 21.63% (Figure S5), closely matching the theoretical value of 21.55% derived from chemical formula analysis. The UV–vis spectrum of Au_75_ in methanol reveals five prominent optical absorption bands at 320, 345, 385, 420, 475, 600, and 715 nm (Figure S6), and the unattenuated absorptions of the nanocluster demonstrate its structural robustness and high stability (Figure S7). In addition, we compared the ^19^F nuclear magnetic resonance (^19^F NMR) spectroscopy of the P­(C_6_H_5_-4-CF_3_)_3_ ligand and fluorine-containing nanoclusters (Au_75_ and Au_11_). As shown in Figure S8, ^19^F NMR spectra of the three samples are located very closely around −64 ppm, demonstrating that the −CF_3_ groups on the nanocluster surface maintain an almost unchanged chemical environment. Indeed, for the spherical Au_75_ cluster structure, all P­(C_6_H_5_-4-CF_3_)_3_ ligands follow the same Au–P interactions.

Single-crystal X-ray diffraction analysis reveals that the Au_75_ cluster molecules crystallize in the trigonal space group *R*-3 with an ABAB stacking arrangement (Figures S9 and S10; Table S1), together with Cl^–^ counterions with a molar ratio of 1:2 (cluster:counterion) in the crystalline lattice. A detailed molecular-level analysis reveals that the cluster comprised 75 gold atoms, 20 P­(C_6_H_4_-4-CF_3_)_3_ ligands, and 12 Cl ligands, resulting in its overall formula of Au_75_(P­(C_6_H_4_-4-CF_3_)_3_)_20_Cl_12_ (Figure S11). As illustrated in Figure 2, the Au_75_ nanocluster adopts a Russian doll-like shell-by-shell configuration: Au_13_@Au_42_@Au_20_@Cl_12_@(PR)_20_. The innermost core of the Au_75_ nanocluster is an Au_13_ icosahedron, surrounded by an Au_42_ McKay icosahedral shell (Figure 2A,B). The first two shells of the Au_75_ nanocluster form an Au_55_ kernel (Figure 2C), aligning with the nuclei number of the well-known “Schmid gold”. The axial thickness of the Au_55_ kernel is 1.10 nm, while the equatorial diameter is 1.23 nm (Figure S12). Furthermore, the Au_55_ kernel is fully encapsulated by 20 peripheral Au atoms, which are arranged in a configuration resembling the smallest fullerene, C_20_;^47^ in this context, this Au_20_ shell is referred to as the “golden fullerene” (Figure 2D,E). Then, the fourth and fifth layers of the Au_75_ cluster consist of 12 chlorine ligands arranged in an icosahedral configuration and 20 phosphine ligands configured similarly to a C_20_ fullerene, respectively, providing an all-around protection for the Au_75_ metallic kernel (Figure 2F–H). The average Au–Au bond lengths in Au_13_ and Au_42_ shells are 2.791 and 2.916 Å, respectively (Table S4). The Au–Au distances between the Au_55_ core and the golden fullerene Au_20_ shell range from 2.718 to 2.769 Å (Table S5). In addition, the average bond lengths at the interface between the metallic kernel and the organic ligand layer are determined to be 2.306 Å for Au–Cl bonds and 2.311 Å for Au–P bonds.

**Figure 2:**
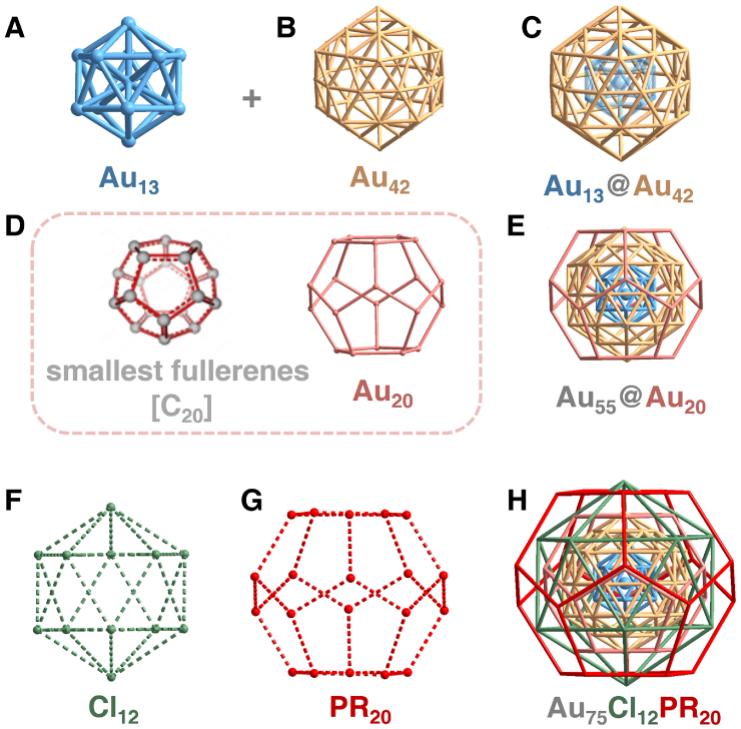
Structure anatomy of the Au_75_ nanocluster. (A) The first shell (labeled in blue): Au_13_ Mackay icosahedron. (B) The second shell (labeled in yellow): Au_42_ Mackay icosahedron. (C) The Au_55_ core. (D) The third shell (labeled in pink): Au_20_ shell with a C_20_ fullerene-like configuration. (E) Metallic structure of the Au_75_ nanocluster. (F) The fourth shell (labeled in green): Cl_12_ icosahedral shell. (G) The fifth shell (labeled in red): (PR)_20_ shell with a C_20_ fullerene-like configuration. (H) Russian doll-like shell-by-shell configuration of the Au_75_ nanocluster.

A detailed structural analysis is then conducted to elucidate how the golden fullerene Au_20_ shell stabilized the Au_55_ kernel. Considering that the Au_55_ kernel contains an inner Au_13_ icosahedral core and an outer Au_42_ Mackay icosahedron shell, we mainly focus on the interactions between the golden fullerene Au_20_ shell and the Au_42_ shell. The Au_42_ Mackay icosahedron comprises 12 vertexes, 30 edges, and 20 faces. As depicted in Figure 3A,B, the 42 shell atoms are classified into two groups: 12 Au atoms located at the vertices and 30 Au atoms along the edges of the Mackay icosahedron. First, each gold atom at vertex positions is positioned at the center of a five-membered Au_5_ face from the golden fullerene Au_20_ shell, corresponding to 12 faces of this fullerene-like shell (Figure 3C). The average bond angle of the Au_1_@Au_5_ ring is 60.98°. In this context, although the overall arrangement displays *C*_5_ symmetry, the sum of angles (304.9°) indicates that the Au_12_ vertex of the Au_55_ kernel slightly protrudes above the golden fullerene Au_20_ shell (Figure 3D). In addition, every three adjacent Au atoms from the edges of the Mackay icosahedron form a tetrahedron together with an Au atom from the golden fullerene Au_20_ shell (Figure 3E). These 20 generated tetrahedra correspond perfectly to the 20 faces of the Mackay icosahedron, fully encapsulating and stabilizing the Au_55_ kernel (Figure 3F). The average Au–Au bond length within these tetrahedra is 2.739 Å, much shorter than the Au–Au distances within Au_13_ and Au_42_ shells, suggesting robust interactions between the golden fullerene Au_20_ shell and its stabilized Au_55_ kernel.

**Figure 3:**
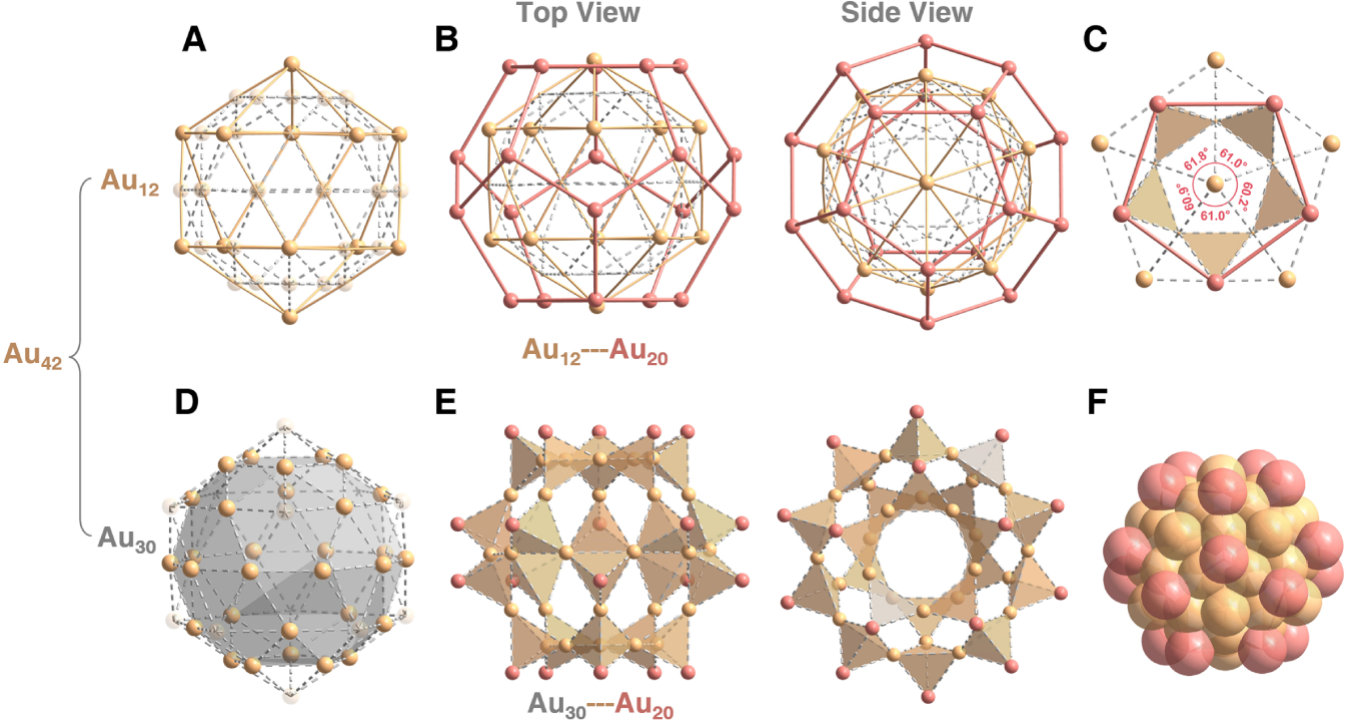
Interactions between the Au_20_ golden fullerenes and the Au_42_ shell. (A) 12 Au atoms locating at vertexes of the icosahedral Au_42_ shell. (B) Each Au atom in Au_12_ situating in the central position of the pentagon from the fullerene-like Au_20_. (C) A typical golden pentagon. (D) 30 Au atoms locating on edges of the icosahedral Au_42_ shell. (E) Every three adjacent Au atoms in Au_30_ forming a tetrahedron with an Au atom from the fullerene-like Au_20_. (F) Space-filling form of the metallic kernel.

Electrospray ionization time-of-flight mass spectrometry (ESI-TOF-MS) in positive mode reveals two dominant signals at m/*z* of 12261.59 and 8331.80 Da, corresponding to the [Au_75_(P­(C_6_H_4_-4-CF_3_)_3_)_20_Cl_12_]^2+^ and its dissociated peak [Au_55_(P­(C_6_H_4_-4-CF_3_)_3_)_12_Cl_6_Na]^2+^, respectively (Figure S13). First, the +2-charge state of the Au_75_ nanocluster matches the presence of twice the molar quantity of Cl^–^ of cluster molecules in the crystal lattice. In this context, the nominal electron count^48^ of the Au_75_ nanocluster is determined as 61, i.e., 75 (Au) – 12 (Cl) – 2 (positive charge) = 61e, suggesting the presence of an unpaired electron within the cluster framework. Electron paramagnetic resonance (EPR) further confirms an open-shell electronic configuration for the Au_75_ nanocluster, displaying a distinct signal with a single local maximum and minimum at *S* = 1/2 (*g* = 1.4078, 2.0314) (Figure S14). Second, the detected [Au_55_(P­(C_6_H_4_-4-CF_3_)_3_)_12_Cl_6_Na]^2+^ molecule is anchored by a Na^+^ in the mass detection environment, leading to the direct observation of the molecule-ion mass signal of the fluorinated “Schmid gold”, [Au_55_(P­(C_6_H_4_-4-CF_3_)_3_)_12_Cl_6_]^+^.

The structural analysis further indicates that the fluorinated ligands introduce rich F···F and H···F interactions at both the Au_75_ nanocluster surface and the intercluster interface (Figure S15). In comparison to the PPh_3_ ligands, the fluorinated ligands enhance the cluster stability by improving interactions between the gold kernel and the ligand shell, facilitating the detection of the Au_75_ nanocluster in mass spectrometry. Furthermore, the abundance of F-initiated interactions can be thought to contribute to greater structural rigidity at the molecular level and promote the long-range ordered arrangement of cluster molecules within the crystal lattice, endowing the Au_75_ nanocluster with good crystallinity. We performed the analysis for the solvent-accessible surface area SASA (see Supporting Information for technical details) for the Au_75_(P­(C_6_H_4_-4-CF_3_)_3_)_20_Cl_12_ cluster, its hypothetical counterpart Au_75_(PPh_3_)_20_Cl_12_, and a hypothetical icosahedral model for the fluorinated “Schmid cluster” Au_55_(P­(C_6_H_4_-4-CF_3_)_3_)_12_Cl_6_ (for visualization of their full atomic structures, see Figure S16). The larger the value of SASA is, the more accessible the gold core surface is for small molecules to penetrate from solvent, hence a zero SASA value means a fully sterically protected metal–ligand interface. By using a typical “small molecule size” of 1.4 Å, we found that both Au_75_(P­(C_6_H_4_-4-CF_3_)_3_)_20_Cl_12_ and Au_75_(PPh_3_)_20_Cl_12_ clusters are fully sterically protected but the hypothetical fluorinated “Schmid cluster” has a large SASA value of 23.3 Å^2^.

We performed density functional theory (DFT) calculations to analyze the electronic structure and evaluate the optical absorption of [Au_75_(P­(C_6_H_4_-4-CF_3_)_3_)_20_Cl_12_]^2+^ (Figure 4A; for technical details of DFT calculations, Supporting Information). Figure 4B shows that the computed UV–vis absorption spectrum compares rather well with the measured data in its overall shape, reproducing a major experimental feature at 600 nm. We also computed the spectrum using a few other charge states of the cluster, including charges of −1, +3, and +5, but found out that the computed spectra did not match the experimental data well (Figure S17). This gives indirect support for the experimentally determined cluster charge of +2.

**Figure 4:**
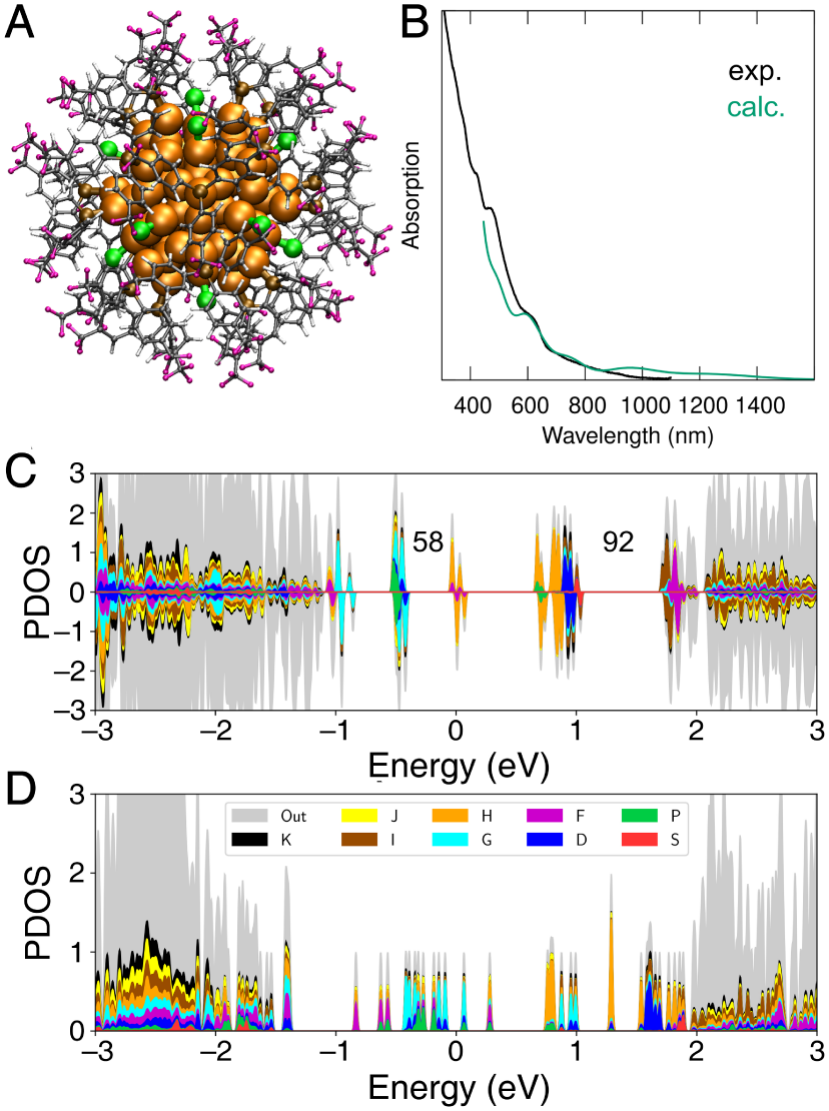
(A) Visualization of the atomic structure of Au_75_(P­(C_6_H_4_-4-CF_3_)_3_)_20_Cl_12_^2+^. Color labels: orange = Au; green = Cl; brown = P; gray = C; purple = F; white = H. (B) Computed and measured UV–vis spectra of the Au_75_ nanocluster. The computed spectrum is scaled to the experimental intensity at 600 nm. (C) Superatom-projected density of electron states PDOS. PDOS for spin-alpha states are shown in top and spin-beta states in the bottom panel. The electron shell closures at 58 and 92 are shown as well. (D) PDOS of the hypothetical fluorinated “Schmid cluster” Au_55_(P­(C_6_H_4_-4-CF_3_)_3_)_12_Cl_6_^+^. The Fermi energy is at zero in (C) and (D).

Figure 4C,D compare the electronic density of states of Au_75_(P­(C_6_H_4_-4-CF_3_)_3_)_20_Cl_12_^2+^ and hypothetical fluorinated “Schmid cluster” [Au_55_(P­(C_6_H_4_-4-CF_3_)_3_)_12_Cl_6_]^+^. The electronic states are projected to spherical harmonics centered at the clusters’ center of mass. One can see that Au_75_(P­(C_6_H_4_-4-CF_3_)_3_)_20_Cl_12_^2+^ has narrow “bands” of electronic states in the frontier region with energies ranging from one eV below to about 2 eV above the Fermi energy. All these states are mainly localized in the gold core and follow a nice succession of expected angular momentum symmetries between 58 and 92 electron shell closing in the sequence: 1S^2^ 1P^6^ 1D^10^ 2S^2^ 1F^14^ 2P^6^ 1G^18^ (58e) 1H^22^ 2D^10^ 3S^2^ (92e) 1I^26^ 2F^14^. Thus, the “superatom” electronic structure^43^ is well established in [Au_75_(P­(C_6_H_4_-4-CF_3_)_3_)_20_Cl_12_]^2+^. On the contrary, the electron shells from 1F to 3S are much more split and mixed in the hypothetical fluorinated “Schmid cluster” [Au_55_(P­(C_6_H_4_-4-CF_3_)_3_)_12_Cl_6_]^+^. We attribute the more shell-like electronic structure of [Au_75_(P­(C_6_H_4_-4-CF_3_)_3_)_20_Cl_12_]^2+^ to its stabilizing Au_20_(P­(C_6_H_4_-4-CF_3_)_3_)_20_ fullerene-like overlayer discussed above.

We studied the stabilizing effect of the overlayer with the fluorinated phosphine by calculating the theoretical detachment energy of one phosphine from the corresponding cluster in (i) Au_75_(P­(C_6_H_4_-4-CF_3_)_3_)_20_Cl_12_ cluster, (ii) its hypothetical counterpart Au_75_(PPh_3_)_20_Cl_12_, and (iii) a hypothetical icosahedral model for the fluorinated “Schmid cluster” Au_55_(P­(C_6_H_4_-4-CF_3_)_3_)_12_Cl_6_ (Figure S15), by using an improved electron–electron exchange-correlation functional (see Supporting Information) taking into account the van der Waals interactions in the ligand layer. The detachment energies for the three clusters are (i) 4.74 eV, (ii) 3.02 eV, and (iii) 2.05 eV. This clearly shows that the hypothetical “Schmid cluster” is least stabilized by the fluorinated phosphine, and among the Au_75_ systems the fluorinated phosphine renders clear extra stability to the metal core as compared to the standard triphenylphosphine.

To investigate the impact of fluorination on ligand shell behavior, we performed molecular dynamics simulations (see technical details in the Supporting Information) to compare the stability and dynamics of the ligand layers in the Au_75_(P­(C_6_H_4_-4-CF_3_)_3_)_20_Cl_12_ and Au_75_(P­(Ph)_3_)_20_Cl_12_ clusters in a solvent environment. Gold–phosphorus interaction was parametrized using DFT procedure (described in Supporting Information and Figure S18). We analyzed several distance-based interactions (Figure 5A–H) and compiled average statistics from three independent 1 μs simulations, including the number of interactions, the fraction of simulation frames in which they occurred, and their lifetimes (Figure 5).

**Figure 5:**
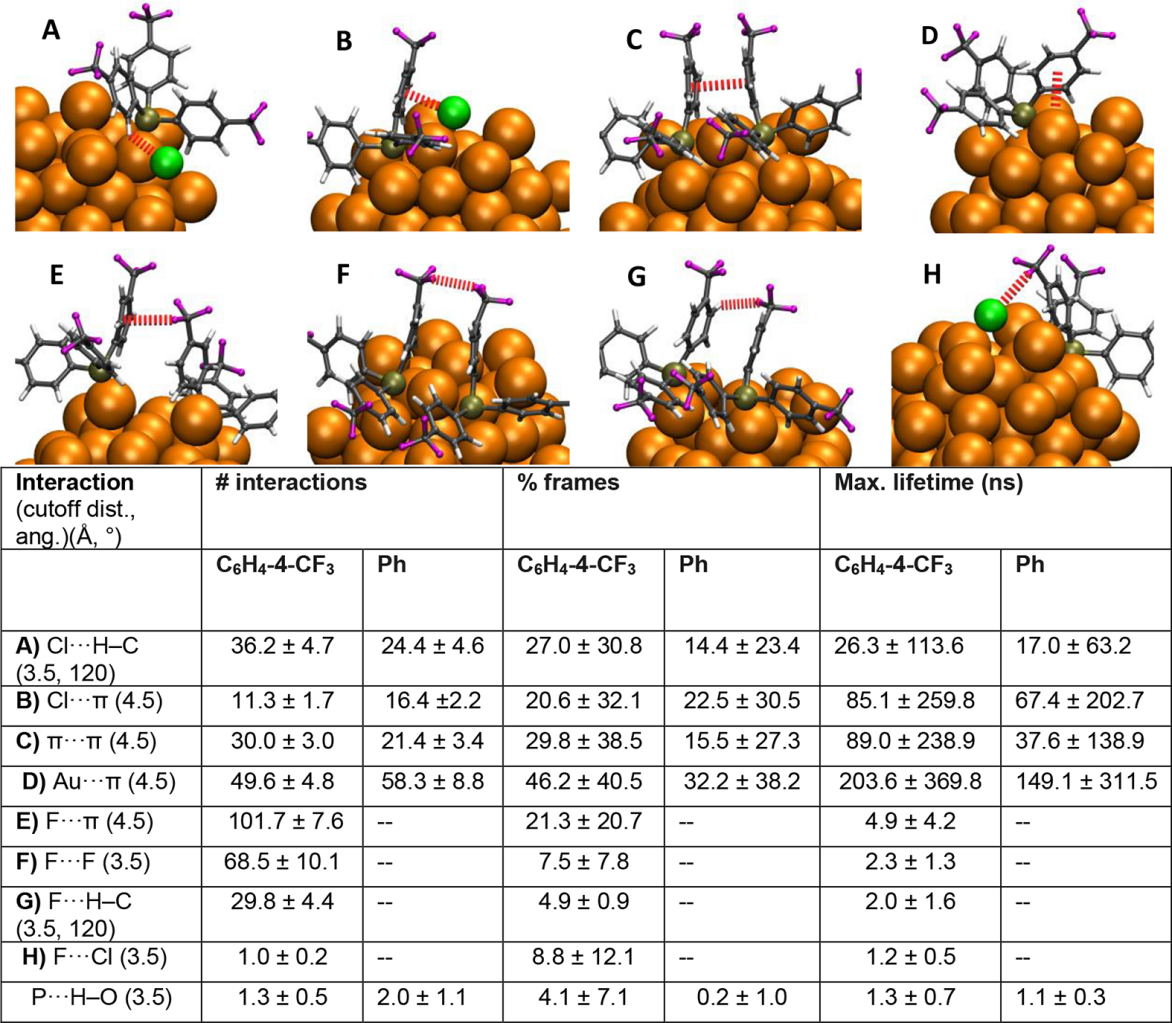
Representative visualizations from molecular dynamics snapshots illustrating intracluster interactions observed in the simulations: (A) Cl···H–C, (B) Cl···π, (C) π···π, (D) Au···π, (E) F···π, (F) F···F, (G) F···H–C, and (H) F···Cl. Cutoff distances and angular criteria used to define these interactions are summarized in the accompanying table. For each interaction type, the table also reports the average number of interactions per frame (±standard deviation), the percentage of frames in which the interaction occurs, and the maximum continuous lifetime of the interaction. These metrics are provided for two cluster systems: Au_75_(P­(C_6_H_4_-4-CF_3_)_3_)_20_Cl_12_ (abbreviated as C_6_H_4_-4-CF_3_) and Au_75_(P­(Ph)_3_)_20_Cl_12_ (abbreviated as Ph). In addition to intracluster interactions the final row of the table shows statistics of interactions with the solvent P···H–O.

Fluorination was found to influence the interactions and enhance stability through two distinct mechanisms: 1) Direct involvement of fluorine in interactions—while clearly present these interactions tend to be more transient, appearing in fewer frames and exhibiting shorter lifetimes compared to interactions not involving fluorine. 2) Stabilization of nonfluorine-mediated interactions—in fluorinated systems, these interactions generally persist longer and are present in more frames than in the nonfluorinated counterpart. Regarding interaction types present in both clusters, Cl···π and Au···π contacts were more prevalent in the nonfluorinated system. This may indicate that the absence of fluorine leads to fewer interphosphine interactions, possibly due to increased ligand flexibility or reduced steric hindrance, allowing ligands to interact more with the cluster surface rather than with each other.

We also assessed interactions between the ligands’ phosphorus atoms and methanol solvent molecules. Although these interactions were infrequent and the differences between systems were minor, the nonfluorinated cluster exhibited slightly more frequent contacts with the solvent. This may suggest a less stable ligand shell in the absence of fluorination.

Finally, we examined the Tolman cone angles,^49^ which characterize the steric bulk of the ligands, for both systems. The cone angles were found to be very similar, 132.2 ± 7.2° and 132.7 ± 6.5° for fluorinated and nonfluorinated clusters, respectively, indicating that fluorination does not significantly influence ligand bulkiness. Instead, the majority of the steric contribution appears to originate from the phenyl rings. Moreover, the variation in cone angles throughout the simulations was relatively small in both cases, suggesting that the ligand bulk remains stable over time and does not undergo substantial dynamic changes.

Since the Au_75_ nanocluster possesses a large Au_55_ core, it would be insightful to probe its excited-state behaviors. The femtosecond transient absorption (fs-TA) spectrum of the Au_75_ nanocluster exhibits distinct features. Under 400 nm excitation (Figure 6A), the excited-state absorption signal decays completely to baseline within 100 ps (Figure 6B), in sharp contrast to the nanosecond-scale lifetimes observed in phosphine-stabilized clusters like Au_11_ (24 ns) and Au_25_ (1442 ns) (Figures 6B, S19, and S20). Global analysis reveals biphasic decay kinetics, comprising two components: 2.67 ps (τ_1_) and 7.33 ps (τ_2_) (Figure 6C). The picosecond lifetime significantly distinguishes Au_75_ from typical molecular nanoclusters (e.g., Au_25_), while it closely aligns with the electron relaxation behavior of large-sized nanoclusters (e.g., Au_144_, τ ∼ 3 ps).^50,51^

**Figure 6:**
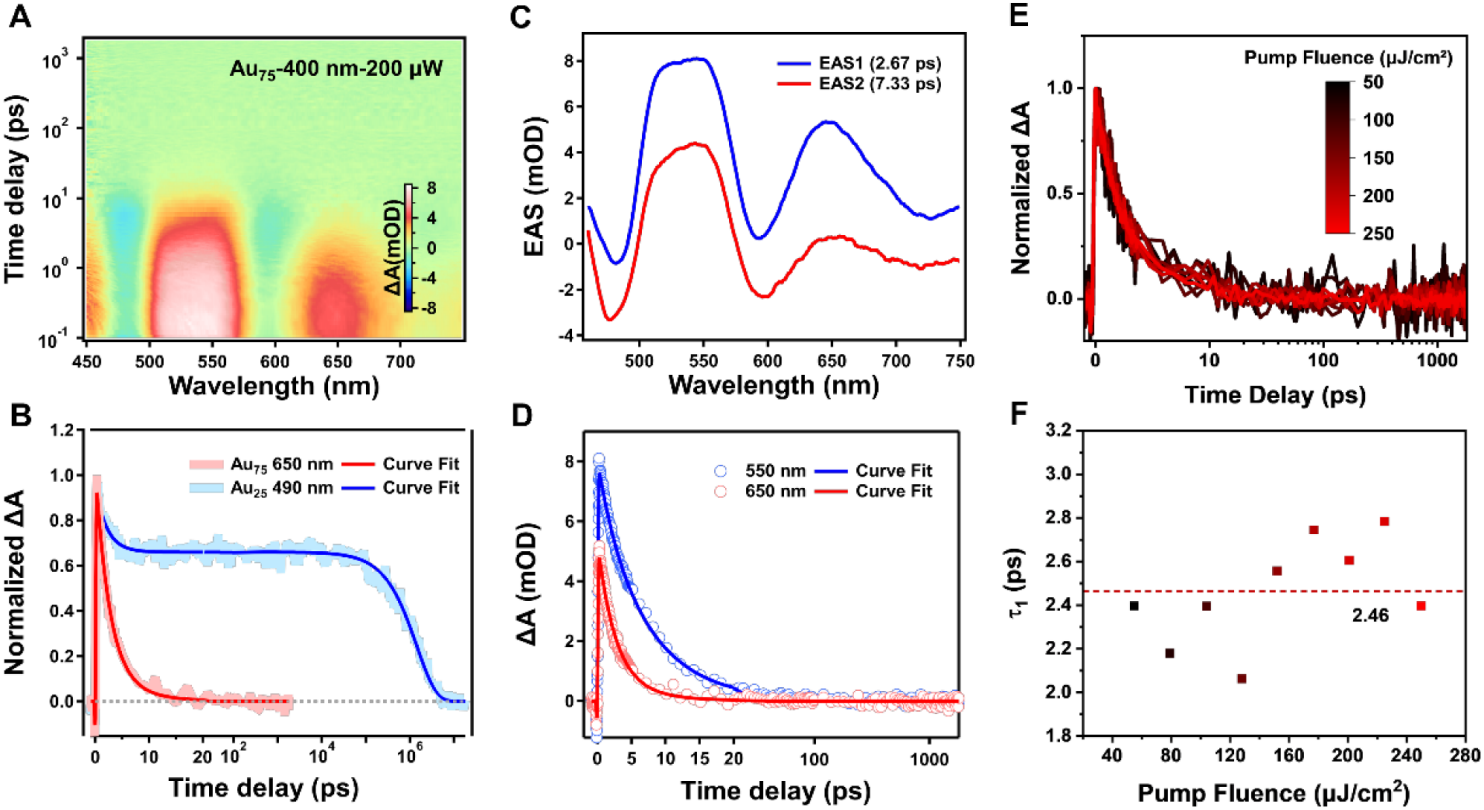
Femtosecond transient absorption dynamics of the Au_75_ nanocluster under 400 nm excitation. (A) fs-TA 2D pseudocolor map of Au_75_ excited at 200 μW (250 μJ/cm^2^). (B) Normalized ESA kinetic traces of Au_75_ (probe at 650 nm) and Au_25_ (probe at 490 nm), combining fs-TA and ns-TA data. (C) EAS obtained from global fitting of the Au_75_ fs-TA data. (D) Kinetic traces and corresponding global fits of Au_75_ at probe wavelengths of 550 and 650 nm. (E) Power-dependent experiment of Au_75_ under 400 nm excitation (54.9–250 μJ/cm^2^), probed at 650 nm. (F) Extracted fast component lifetime τ_1_ from biexponential fitting at 650 nm as a function of excitation power.

Differential pulse voltammetry measures the HOMO–LUMO gap of Au_75_ to be 1.58 eV (Figure S21), but its short lifetime suggests “metallic-like” electronic properties induced by the Au_55_ core (diameter 1.23 nm). The fast decay component (τ_1_ = 2.67 ps) exhibits a time scale similar to the electron–phonon (e-ph) coupling process in metallic nanoclusters.^52^ To understand its nature, we performed TA experiments under different pump fluences (54.9–250 μJ/cm^2^, 400 nm excitation; Figures S22 and S23). As shown in Figure 6E, the normalized TA kinetic traces probed at 650 nm remain unchanged at different power fluences. The fitted τ_1_ values stabilize at 2.46 ± 0.36 ps (Figure 6F), showing no significant power dependence, which indicates that its electron relaxation behavior retains molecular-like features.^53^ This finding is consistent with the absence of a surface plasmon resonance (SPR) peak in the UV–vis absorption spectrum of Au_75_ (Figure S6), which differs from the typical behavior observed in metallic nanoclusters like Au_279_ and aligns with the characteristics of nonmetallic clusters.^54^ Further validation through excitation at 500 and 600 nm reveals that τ_1_ decay component remains at all excitation wavelengths (Figure S24), with decay rates remaining unchanged, effectively excluding the contribution of intramolecular conversion (IC) processes.^51,55^

Notably, the global fitting of the excited-state absorption spectra (EAS) shows similar spectral profiles for both τ_1_ and τ_2_ components (Figure 6C), indicating that the higher excited-state cooling and recombination processes show similar spectral features. The quality of the global fitting is further confirmed at representative probe wavelengths (550 and 650 nm), where the fitted curves closely match the experimental kinetic traces (Figure 6D). This “non-spectral evolution” characteristic is typical for large metallic nanoclusters, such as Au_333_, which can be attributed to the strong electronic coupling between the Au_55_ core and the Au_20_ fullerene shell (average Au–Au bond length of 2.739 Å). The strong core–shell coupling provides an efficient relaxation channel for hot electrons, thereby imparting a unique “size-dependent transitional” dynamic behavior to Au_75_.

As a medium-sized nanocluster, Au_75_ exhibits unique excited-state dynamics that transcend conventional size classifications. Its biphasic decay kinetics share similar features with larger metallic clusters: picosecond-scale lifetimes and temporally overlapping cooling/recombination processes manifest as “non-spectral evolution”, reminiscent of behaviors in systems like Au_333_.^52^ Yet simultaneously, Au_75_ retains molecular-like features including pump-fluence-independent relaxation and absence of SPR-traits characteristic of small quantum-confined clusters. This dual behavior is particularly striking when compared to similarly sized counterparts: the subnanosecond decay in Au_75_ contrasts sharply with nanosecond-scale lifetime in Au_64_.^55^ The rapid excited-state relaxation in Au_75_ can be attributed to the Russian doll architecture, where strong electronic coupling at the Au_55_ core/Au_20_ fullerene interface (evidenced by shortened 2.739 Å bonds) creates efficient relaxation pathways. However, the curved fullerene topology imposes geometric confinement that suppresses electron delocalization, preserving the 1.58 eV HOMO–LUMO gap and preventing metallic bond formation. This fundamentally contrasts with the hybrid electronic structure in Au_156_ where dense energy bands enable partial metallization.^53^ Collectively, these findings demonstrate that geometric constraints dominate over size effects in dictating electron relaxation pathways in Au_75_, establishing a new design principle for tailoring nanocluster photodynamics.

## Conclusion

In summary, we reported the integration of the cluster chemistry with fluorine chemistry, which generated the largest structurally resolved nanocluster protected by phosphine ligands, Au_75_(P­(C_6_H_4_-4-CF_3_)_3_)_20_Cl_12_, whose geometric structure could be likened as an Au_20_ golden fullerene encapsulating the “Schmid gold”. The reported Au_75_ nanocluster followed a Russian doll-like shell-by-shell configuration of Au_13_@Au_42_@Au_20_@Cl_12_@PR_20_, wherein the first-second shell resembled the metallic kernel of the “Schmid gold”, and the third shell showcased a fullerene-like topology, similar to the smallest fullerene C_20_. DFT analysis shows the superatom character of the fluorinated Au_75_ cluster and the stabilizing effect of the fullerene-like overlayer on the electronic structure and chemical stability of the fluorinated Au_75_ nanocluster. Molecular dynamics simulations confirm that fluorination enhances ligand-shell stability in Au_75_ clusters by mediating transient fluorine-involved interactions, reducing solvent exposure and promoting longer-lived nonfluorine-mediated contacts. The femtosecond transient absorption analysis revealed that geometric constraints—specifically, the golden fullerene encapsulating Schmid gold—dominate over size effects in dictating electron relaxation pathways in Au_75_, establishing a new design principle for tailoring the photodynamics of nanoclusters. Collectively, we demonstrate that the introduction of fluorine chemistry to the cluster study carries significant importance. The fluorination ushers in a new era for the fabrication of novel nanoclusters with strong stability and good crystallinity, thus heralding a significant step forward in their downstream applications.

## Methods

### Synthesis of Au_75_

A solution consisting of HAuCl_4_·3H_2_O and P­(C_6_H_4_-4-CF_3_)_3_ was prepared by dissolving these compounds in a mixed solvent of methanol and dichloromethane. After vigorous stirring for 15 min, cyclohexyl mercaptan was added. The solution was then stirred for 30 min, after which NaBH_4_ aqueous solution was added dropwise. The reaction was proceeded at room temperature for 10 h. After this, the solution was centrifuged to remove insoluble precipitates. The supernatant was evaporated to dryness, and the resulting product was washed with *n*-hexane, giving rise to the Au_75_ nanocluster. High-quality crystals of the Au_75_ nanocluster were obtained by diffusing *n*-hexane into the dichloromethane solution containing the nanocluster over 1 week.

### Computational Details

The Density Functional Theory calculations were done by using the GPAW software.^56^ The molecular dynamics simulations were done by using the GROMACS software.^57^ See Supporting Information for computational details.

## Supplementary Material



## Data Availability

All data supporting the findings of this study are included within the article and its Supporting Information. Crystallographic data for the structures reported in this article have been deposited at the Cambridge Crystallographic Data Center, under deposition numbers CCDC 2429951 (Au_75_), 2442163 (Au_25_), and 2442020 (Au_11_). Copies of the data can be obtained free of charge via https://www.ccdc.cam. ac.uk/structures/. Source data are provided with this paper.

## Notes

Detailed discussion on experimental and computational methods (Figures S1–S23 and Tables S1–S5) (PDF)
