# Supplementary material for: A Golden Fullerene Encapsulating Schmid Gold
Source: J Am Chem Soc. 2026 Jan 15;148(4):4579–87. doi: 10.1021/jacs.5c20164 (PMC12879730; doi:10.1021/jacs.5c20164)
Supplement: Supplementary file 1 [file ja5c20164_si_001.pdf]

Supporting Information

A Golden Fullerene Encapsulating Schmid Gold

Peiyao Pan, Sami Malola, Rui Zhao, Wentao Huang, Emmi Pohjolainen, María Francisca Matus, Meng Zhou,* Xi Kang,* Hannu Häkkinen,* Manzhou Zhu*

Content

1. Experimental Section

1.1 Chemicals

1.2 Preparation of the Au_{75} nanocluster

1.3 Preparation of the Au_{11} nanocluster

1.4 Preparation of the Au_{25} nanocluster

1.5 Measurements

1.6 Single Crystal Analysis

1.7 Femtosecond and nanosecond transient absorption

2. Computational methods

2.1 DFT calculations

2.2 Molecular dynamics simulations

3. Supplementary Figures and Tables

Supplementary Figure S1 to S24

Supplementary Table S1 to S5

1. Experimental Section

1.1 Chemicals

Tetrachloroauric(III) acid ($HAuCl_4·3H_2O$, 99.99% metals basis, Aladdin), tris(4-trifluoromethylphenyl)phosphine [$P(C_6H_4\text{-}4\text{-}CF_3)_3$, 97%, Aladdin], cyclohexyl mercaptan (99.99%, Aladdin), triphenylphosphine (PPh_3, 99.9%, Aladdin), sodium borohydride ($NaBH_4$, 98%, Aladdin), methanol (MeOH, 99.9%, Aladdin), dichloromethane (CH_2Cl_2, 99.9%, Aladdin), and *n*-hexane (HPLC, Aladdin). All reagents employed were commercially available and used as received.

1.2 Preparation of the Au_{75} nanocluster

A solution consisting of 300 µL of $HAuCl_4·3H_2O$ (0.2 g/mL) and 50 mg of $P(C_6H_4\text{-}4\text{-}CF_3)_3$ was prepared by dissolving these compounds in a mixed solvent of methanol (CH_3OH) and dichloromethane (CH_2Cl_2). After vigorous stirring for 15 minutes, 100 µL of cyclohexyl mercaptan was added to the mixture. The solution was then stirred for 30 minutes, after which 1 mL of a freshly prepared $NaBH_4$ aqueous solution (2 mg/mL) was added dropwise while stirring vigorously. The color of the solution changed rapidly from light yellow to dark brown. The reaction was proceeded at room temperature for 10 hours under continuous stirring. After this, the solution was centrifuged to remove insoluble precipitates. The supernatant was evaporated to dryness, and the resulting product was washed five times with *n*-hexane, giving rise to the Au_{75} nanocluster with a high purity. High-quality crystals of the Au_{75} nanocluster were obtained by diffusing *n*-hexane into the dichloromethane solution containing the nanocluster over one week. The yield of Au_{75} crystals was approximately 16.7% based on Au.

1.3 Preparation of the Au_{11} nanocluster

The method for synthesis of $Au_{11}(P(C_6H_4\text{-}4\text{-}CF_3)_3)_7Cl_3$ was similar to that for the $Au_{75}(P(C_6H_4\text{-}4\text{-}CF_3)_3)_{20}Cl_{12}$ nanocluster. The only difference was that cyclohexyl mercaptan was absent. Red block crystals were crystallized from CH_2Cl_2/hexane at room temperature after 4 d. The yield of Au_{11} crystals was approximately 20.1% based on Au.

1.4 Preparation of the Au_{25} nanocluster

The synthesis method for $Au_{25}(PPh_3)_{10}(SC_6H_{11})_5Cl_2$ was similar to that used for the $Au_{75}(P(C_6H_4\text{-}4\text{-}CF_3)_3)_{20}Cl_{12}$ nanocluster. The only difference was that 50 mg of $P(C_6H_4\text{-}4\text{-}CF_3)_3$ was substituted with 50 mg of PPh_3. Black block crystals were crystallized from CH_2Cl_2/hexane at room temperature after 5 d. The yield of Au_{25} crystals was approximately 13.26% based on Au.

1.5 Measurements

UV-Vis-NIR absorption spectrum was recorded on an Agilent 8453 diode array spectrometer

Electrospray ionization (ESI) mass spectra were recorded on a Bruker impact II high-definition mass spectrometer, quadrupole, and time-of-flight (Q/TOF) modules in the positive ion mode. The sample was dissolved in CH_3OH.

Thermogravimetric analysis (TGA) was carried out using a thermogravimetric analyzer (DTG-60H, Shimadzu Instruments, Inc.).

X-ray photoelectron spectroscopy (XPS) measurements were performed on a Thermo ESCALAB 250 configured with a monochromatized Al Kα (1486.8 eV) 150 W X-ray source, 0.5 mm circular spot size, flood gun to counter charging effects, and analysis chamber base pressure lower than 1

× 10^{-9} mbar.

Electron paramagnetic resonance (EPR) spectra were collected on a Bruker EMX plus 10/12 (equipped with Oxford ESR910 Liquid Helium cryostat), at the High Magnetic Field Laboratory (Hefei City), Chinese Academy of Sciences.

1.6 Single Crystal Analysis

The data collection for single-crystal X-ray diffraction (SC-XRD) of all nanocluster crystal samples was carried out on a Stoe Stadivari diffractometer under nitrogen flow using a graphite-monochromatized Cu Kα radiation source (λ = 1.54186 Å). Data reductions and absorption corrections were performed using the SAINT and SADABS programs, respectively. The structure was solved by direct methods and refined with full-matrix least squares on F^2 using the SHELXTL software package. All nonhydrogen atoms were refined anisotropically, and all hydrogen atoms were set in geometrically calculated positions and refined isotropically using a riding model. All crystal structures were treated with PLATON SQUEEZE, and the diffuse electron densities from residual solvent molecules were removed. The CCDC numbers of Au_{75}, Au_{11}, and Au_{25} nanoclusters are 2429951, 2442163, and 2442020, respectively.

1.7 Femtosecond and nanosecond transient absorption

Femtosecond transient absorption (fs-TA) measurements were conducted using a home-built pump-probe spectrometer based on a regeneratively amplified Ti:sapphire laser system (800 nm, 35 fs, 7.5 mJ pulse energy, 1 kHz repetition rate, Coherent). The pump pulse was generated via a commercial collinear optical parametric amplifier (Light Conversion), while a portion of the fundamental 800 nm pulse was focused onto a sapphire plate (Eskma) to generate a white light continuum (WLC) spanning 420–800 nm. The WLC was split into signal and reference channels by a 50/50 beam splitter. The pump and probe beams were spatially and temporally overlapped, with their time delay controlled by a mechanical translation stage (DL325, Newport) over a range from –1 ps to 2020 ps. Both beams were set at the magic angle (54.7°) using a half-wave plate to eliminate anisotropic contributions and capture purely isotropic dynamics. Transient signals were recorded with dual spectrometers equipped with 2048-element linear detectors (2048CL, Avantes) at a 2 kHz readout rate, yielding an instrument response function (IRF) of ~100 fs.

For fs-TA measurements, Au_{75} was dissolved in methanol, while Au_{11} and Au_{25} were measured in dichloromethane (DCM), using a 1 mm quartz cuvette. Unless specified otherwise, 400 nm excitation was applied, with 500 nm and 600 nm accessed via the OPA's tunable output. The fs-TA data of Au_{75} under 400 nm excitation (200 µW) were globally analyzed using Glotaran software with a multiexponential decay model.

Nanosecond transient absorption (ns-TA) measurements were conducted using the same ultrafast pump pulses along with an electronically delayed supercontinuum light source with a sub-nanosecond pulse duration (Nano100, Time-Tech Spectra). The time resolutions of fs-TA and ns-TA were approximately 100 fs and 1 ns, respectively.

2. Computational methods

2.1 DFT calculations

The DFT calculations were done using the software GPAW.[S1,S2] We used a real space grid with 0.2 Å grid spacing and Perdew-Ernzerhof-Burke (PBE)[S3] electronic exchange correlation functional for

the structure optimization. Optimizations were done until the forces of atoms were below 0.05 eV/Å. The electronic structure analysis was done using the GLLB-sc functional[S4] and 0.3 Å grid spacing. The starting structure of Au_{75} cluster with fluorinated $P(PhCF_3)_3$ ligands was taken from the crystal structure. For comparison, a reference structure of Au_{75} cluster with triphenylphosphine ligands was created using the same crystal structure but replacing $-CF_3$ end groups with -H. A model structure of the "Schmid Au_{55} cluster" was created using icosahedrally symmetric Au_{55} inner core of Au_{75} cluster. In the Au_{55} cluster, fluorinated phosphine ligands were placed at the corners of the Au_{55} icosahedron and the six chlorides at the center of the six (out of total 20) facets of the outermost icosahedral Au_{42} shell. The electronic structure and optical absorption spectrum were done as spin-polarized calculations for Au_{75} with fluorinated ligands using the experimental structure directly with +2 total charge having one unpaired electron. The other studied charge states for Au_{75} cluster with fluorinated ligands were -1, +3 and +5 for which absorption spectra were calculated for comparison using the experimental structure directly. The electronic structure of Au_{55} cluster was calculated with GLLB-sc functional using the PBE-optimized structure and total charge 1+. In all cases the electron density of states was projected to the spherical harmonics functions centered at the center of mass of the cluster inside a cut off sphere of 16.5Å for Au_{75} cluster and 13.5 Å for Au_{55} cluster.[S5] For Au_{75} with +2 charge the projected density of states is represented separately for spin up and spin down states. Absorption spectra of Au_{75} cluster with fluorinated ligands and different charge states were calculated using the experimental structure, GLLB-sc functional for wavefunctions, and linear response time-dependent density functional theory (LR-TDDFT)[S6] with PBE functional as a xc-kernel. The detachment energies of phosphine ligands were calculated using fully PBE-optimized structures of Au_{75} and Au_{55} clusters for selected representative ligands. Energies are given for both PBE and BEEF-vdW functionals.[S7] The BEEF-vdW functional is known to represent the weak van der Waals interactions between ligands in a more realistic way as compared to the PBE functional. The solvent accessible surface area SASA was determined with the VMD software[S8] for the PBE optimized structures of Au_{75} cluster with fluorinated and triphenylphosphine ligands and for Au_{55} cluster with fluorinated ligands. We used a 1.4 Å spherical probe that corresponds in size roughly to the size of water molecule. SASA values indicate the free open surface area of metal core after taking into account the ligands which protect most of the metal core surface.

DFT calculations were also used to obtain the non-bonded (van der Waals) parameters for Au-P interactions. A starting structure of Au_{75} cluster with phosphine ligands (PH_3) was built based on the optimized-crystal structure of $Au_{75}(P(C_6H_4\text{-}4\text{-}CF_3)_3)_{20}Cl_{12}$ but replacing the $-C_6H_4\text{-}4\text{-}CF_3$ groups with -H, and its geometry optimization was done by using the same conditions as previously described. Then, a single representative PH_3 ligand was selected from the nanocluster surface to study the energy behavior around the equilibrium position. This was done by performing several single-point calculations, varying the Au-P bond distance outward and inward from the metal core in 0.10 Å intervals (from 1.85 Å to 3.30 Å). Finally, the Lennard-Jones 12-6 potential was fitted to the DFT energies obtained at each point to determine the ε and σ parameters for molecular dynamics simulations. Data points between 2.15 - 3.0 Å were selected for the best fit.

2.2 Molecular dynamics simulations

Molecular dynamics simulations of $Au_{75}(P(C_6H_4\text{-}4\text{-}CF_3)_3)_{20}Cl_{12}$ and $Au_{75}(P(C_6H_4)_3)_{20}Cl_{12}$ were

performed using Gromacs 2023.3[S9] software with Amber99sb-ildn force field.[S10] Force field parameters for the two phosphine ligands were derived using Ambertools[S11] and converted to Gromacs format using acpype code[S12]. The gold–phosphorous interaction was described with Lennard-Jones 12-6 potential with parameters ε = 140.412 kJ/mol and σ = 0.206 nm derived in the previous section 2.1. Standard non-bonded potentials were used between gold and other atom types, with Lennard-Jones parameters for gold ε = 22.133 kJ/mol and σ = 0.263 nm. [S13] Gold-chlorine interactions were described using covalent and non-covalent bonded parameters described in reference S14. Each system was solvated in methanol[S15] and simulated in periodic cubic box with 2.0 nm distance to the box boundary. Steepest descent energy minimization, 10 ns NVT (300 K) followed by 10 ns NPT (300 K, 1 bar) equilibrations were performed with position restraints applied for all the heavy atoms in the cluster, with force constants of 10000 $kJ \cdot mol^{-1} \cdot nm^{-2}$ for Au atoms and 1000 $kJ \cdot mol^{-1} \cdot nm^{-2}$ for other atom types in xyz-directions. The production runs were performed under NPT conditions using velocity-rescale thermostat (reference temperature 300 K, time constant 0.1 ps) and C-rescale barostat[S16] (reference pressure 1 bar, time constant 2 ps) keeping the position restraints on gold atoms to retain the cluster core geometry, while releasing the position restraints of phosphines. Lengths of bonds containing hydrogen atoms were constrained using LINCS[S17] algorithm. The particle mesh Ewald method[S18] was used to calculate electrostatic interactions with a cut-off length of 1 nm and a grid spacing of 0.12 nm. Van der Waals interactions were cut off at 1 nm with potential-shift-Verlet -modifier and dispersion corrections were applied for energy and pressure. For each system three replicate production simulations of 1 µs were performed starting with random initial velocities.

References

[S1] Mortensen, J. J. et al. GPAW: An open Python package for electronic structure calculations. *J. Chem. Phys.*, **160**, 092503 (2024).

[S2] Enkovaara, J et al. Electronic structure calculations with GPAW: a real-space implementation of the projector augmented-wave method. *J. Phys.: Condens. Matter* **22**, 253202 (2010).

[S3] Perdew J. P., Burke K., Ernzerhof M. Generalized Gradient Approximation Made Simple. *Phys. Rev. Lett.* **77**, 3865-3868 (1996).

[S4] Kuisma, M., Ojanen, J., Enkovaara, J., Rantala, T. T. Kohn-Sham potential with discontinuity for band gap materials. *Phys. Rev. B.* **82**, 115106 (2010).

[S5] Walter, M., et al. A Unified View of Ligand-Protected Gold Clusters as Superatom Complexes. *Proc. Natl. Acad. Sci. U.S.A.* **105**, 9157-9162 (2008).

[S6] Walter, M. et al. Time-dependent density-functional theory in the projector augmented-wave method. *J. Chem. Phys.* **128**, 244101 (2008).

[S7] J. Wellendorff, K. T. et al. Density functionals for surface science: Exchange-correlation model development with Bayesian error estimation. *Phys. Rev. B* **85**, 235149 (2012).

[S8] Humphrey, W., Dalke, A. and Schulten, K. VMD - visual molecular dynamics. *J. Molec. Graphics* **14**, 33-38 (1996).

[S9] Abraham, M. J.; Murtola, T.; Schulz, R.; Páll, S.; Smith, J. C.; Hess, B.; Lindahl, E. GROMACS: High Performance Molecular Simulations through Multi-Level Parallelism from Laptops to Supercomputers. *SoftwareX* **1**, 19–25 (2015).

[S10] Lindorff-Larsen, K., Piana, S., Palmo, K., Maragakis, P., Klepeis, J. L., Dror, R. O. & Shaw, D. E.

Improved side-chain torsion potentials for the AMBER ff99SB protein force field. *Proteins* **78**, 1950–1958 (2010).
[S11] Case, D. A., Cerutti, D. S., Cheatham, T. E., Darden, T. A., Duke, R. E., Giese, T. J., Gohlke, H., Goetz, A. W., Greene, D., Homeyer, N., Izadi, S., Kovalenko, A., Lee, T. S., LeGrand, S., Li, P., Lin, C., Liu, J., Luchko, T., Luo, R., Mermelstein, D., Merz, K. M., Monard, G., Nguyen, H., Nguyen, H. T., Omelyan, I., Onufriev, A., Pan, F., Qi, R., Roe, D. R., Roitberg, A., Sagui, C., Simmerling, C. L., Botello-Smith, W. M., Swails, J., Walker, R. C., Wang, J., Wolf, R. M., Wu, X., Xiao, L. & Kollman, P. A. AmberTools16. University of California, San Francisco (2016).
[S12] Da Silva, A. W. S. & Vranken, W. F**.** ACPYPE – Antechamber Python Parser Interface. *BMC Res. Notes* **5**, 367 (2012).
[S13] Heinz, H., Vaia, R. A., Farmer, B. L. & Naik, R. R. Accurate simulation of surfaces and interfaces of face-centered cubic metals using 12–6 and 9–6 Lennard-Jones potentials. *J. Phys. Chem. C* **112**, 17281–17290 (2008).
[S14] Matus, M. F., Sabooni Asre Hazer, M., Malola, S. & Häkkinen, H. Development of an AMBER-compatible force field for gold nanoclusters protected by N-heterocyclic carbenes. *J. Chem. Theory Comput.* **21**, 12121—12132 (2025).
[S15] Fischer, N. M., van Maaren, P. J., Ditz, J. C., Yildirim, A. & van der Spoel, D. Properties of organic liquids when simulated with long-range Lennard-Jones interactions. *J. Chem. Theory Comput.* **11**, 2938–2944 (2015).
[S16] Bernetti, M. & Bussi, G. Pressure control using stochastic cell rescaling. *J. Chem. Phys.* **153**, 114107 (2020).
[S17] Hess, B., Bekker, H., Berendsen, H. J. C. & Fraaije, J. G. E. M. LINCS: a linear constraint solver for molecular simulations. *J. Comput. Chem.* **18**, 1463–1472 (1997).
[S18] Darden, T., York, D. & Pedersen, L. Particle mesh Ewald: an N·log(N) method for Ewald sums in large systems. *J. Chem. Phys.* **98**, 10089–10092 (1993).

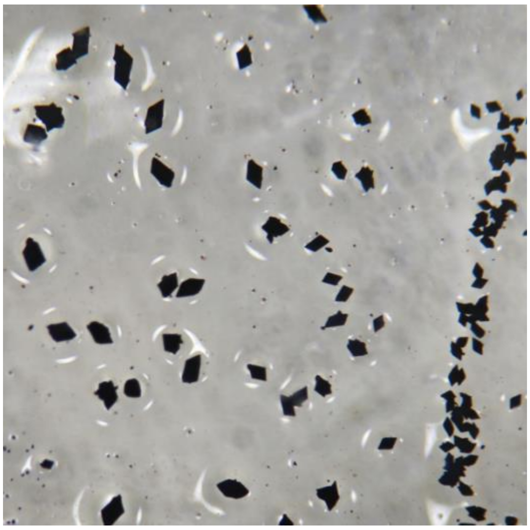

Figure S1. Digital photo of single crystals of the Au_{75} nanocluster, which were cultivated by diffusing *n*-hexane into the dichloromethane solution containing the nanocluster over one week.

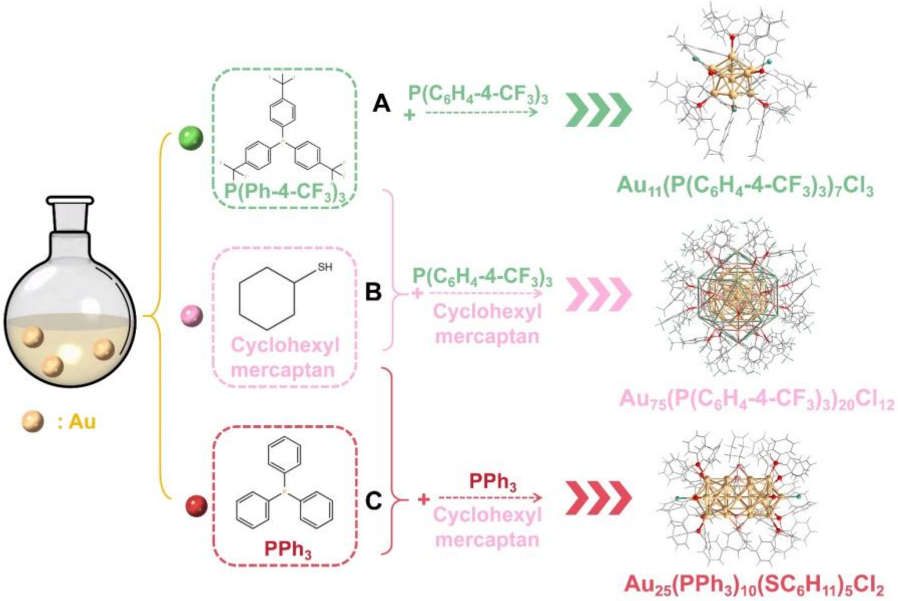

Figure S2. Illustration of the syntheses of Au_{11}, Au_{25}, and Au_{75} nanoclusters.

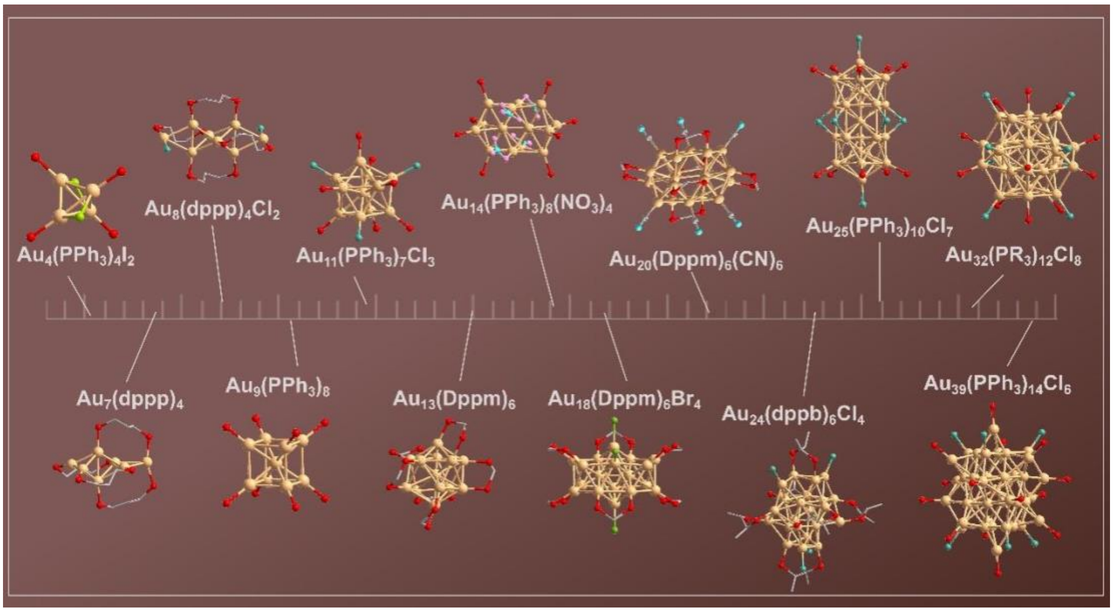

Figure S3. Summary of the reported structurally resolved gold nanoclusters stabilized by phosphine ligands.

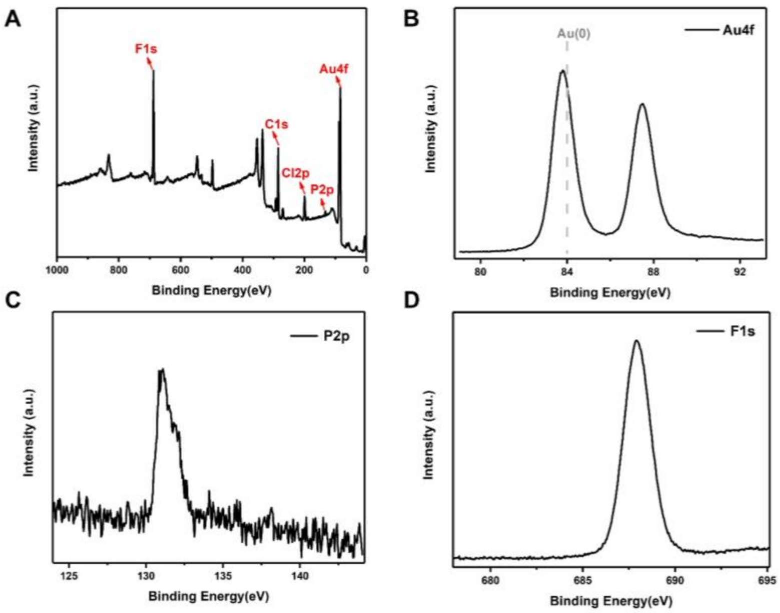

Figure S4. X-ray photoelectron spectroscopy of the Au_{75} nanocluster. (A) XPS full spectrum. (B) Au 4f bands of the Au_{75} nanocluster. (C) P 2P bands of the Au_{75} nanocluster. (D) F 1s bands of the Au_{75} nanocluster. X-ray photoelectron spectroscopy (XPS) analysis demonstrates an F 1s binding energy of 687.9 eV, which is characteristic of the $-CF_3$ substituents in the $P(C_6H_4\text{-}4\text{-}CF_3)_3$ ligands, with no discernible contributions from impurity species. Furthermore, XPS analysis reveals a P 2p binding energy of 132 eV, indicating electron donation from the phosphorus lone pairs to the gold atoms on the cluster surface, and a concomitant decrease in the electron cloud density surrounding phosphorus.

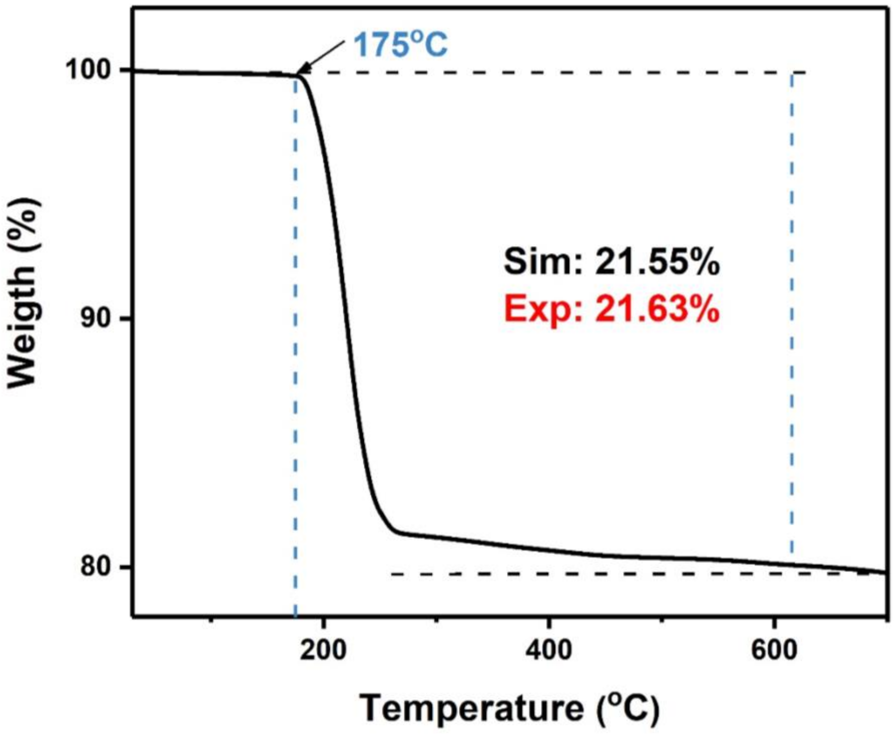

Figure S5. Thermogravimetric analyses curves of the Au_{75} nanocluster. The simulated 21.55% corresponds to phosphine and chlorine ligands that are lost from the surface of the Au_{75} nanocluster

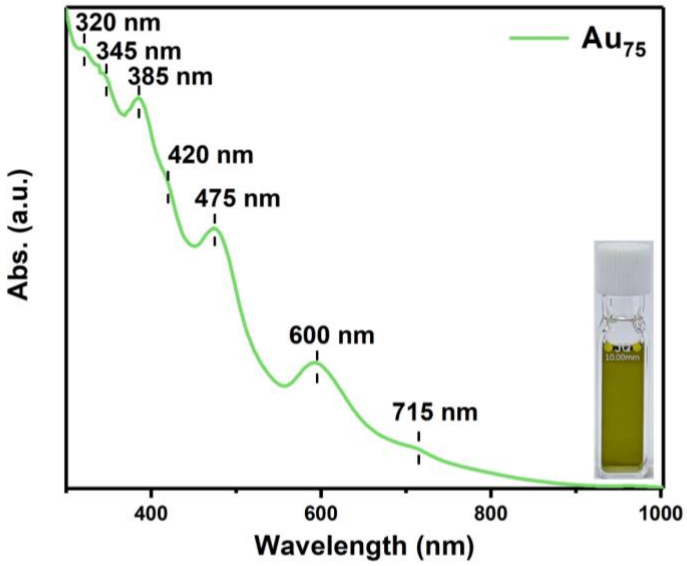

Figure S6. UV-vis spectrum of the Au_{75} nanocluster.

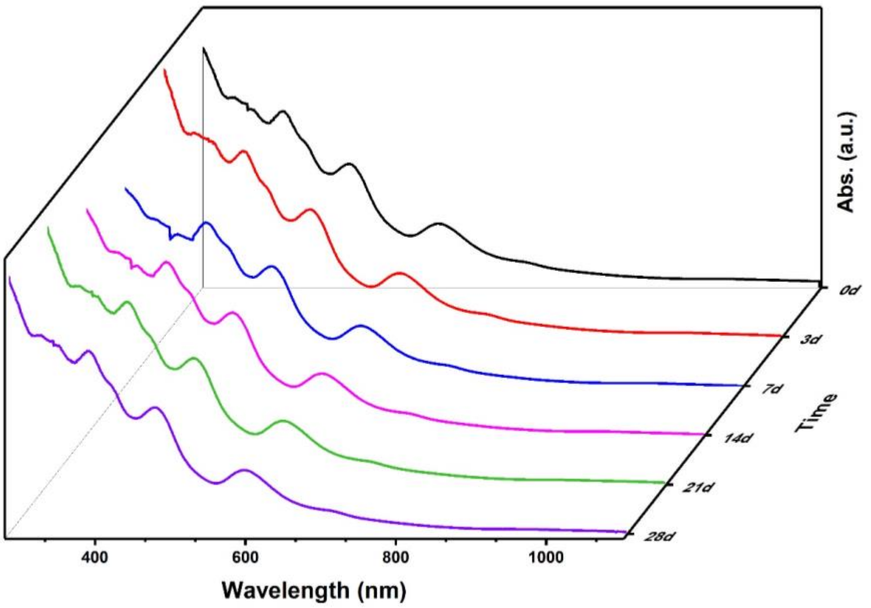

Figure S7. Time-dependent UV-vis spectra of the Au_{75} nanocluster for 28 days.

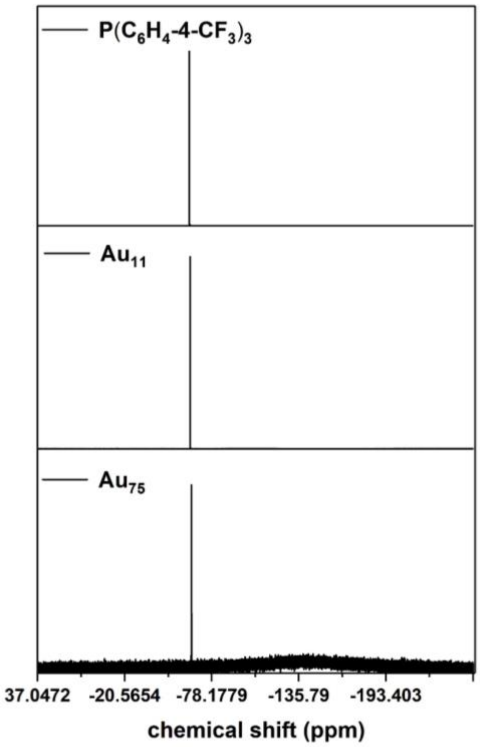

Figure S8. ^{19}F NMR spectra of the $P(C_6H_4\text{-}4\text{-}CF_3)_3$ ligand, the Au_{11} nanocluster, and the Au_{75} nanocluster at -64.32, -64.80, and -64.72 ppm, respectively.

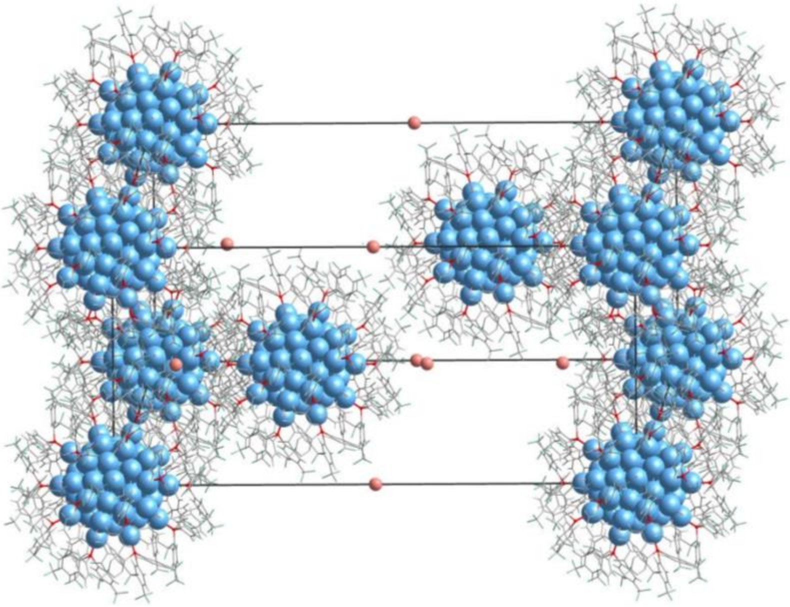

Figure S9. Unit cell of the $[Au_{75}(P(C_6H_4\text{-}4\text{-}CF_3)_3)_{20}Cl_{12}]Cl_2$ cluster molecules. Color legends: blue, Au; pink, Cl; red, P; cyan, F; grey, C; light gray, H.

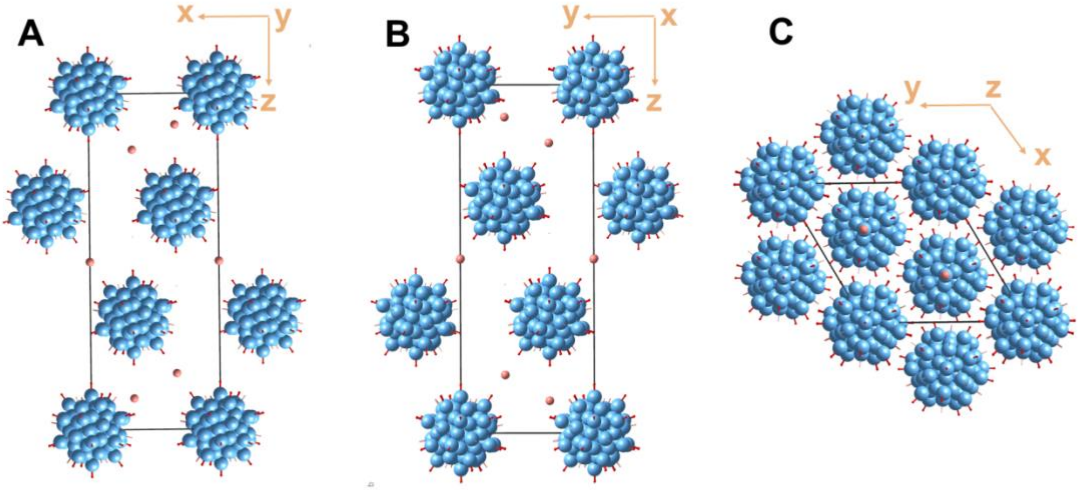

Figure S10. Packing pattern of the $[Au_{75}(P(C_6H_4\text{-}4\text{-}CF_3)_3)_{20}Cl_{12}]Cl_2$ cluster in the crystal lattice.

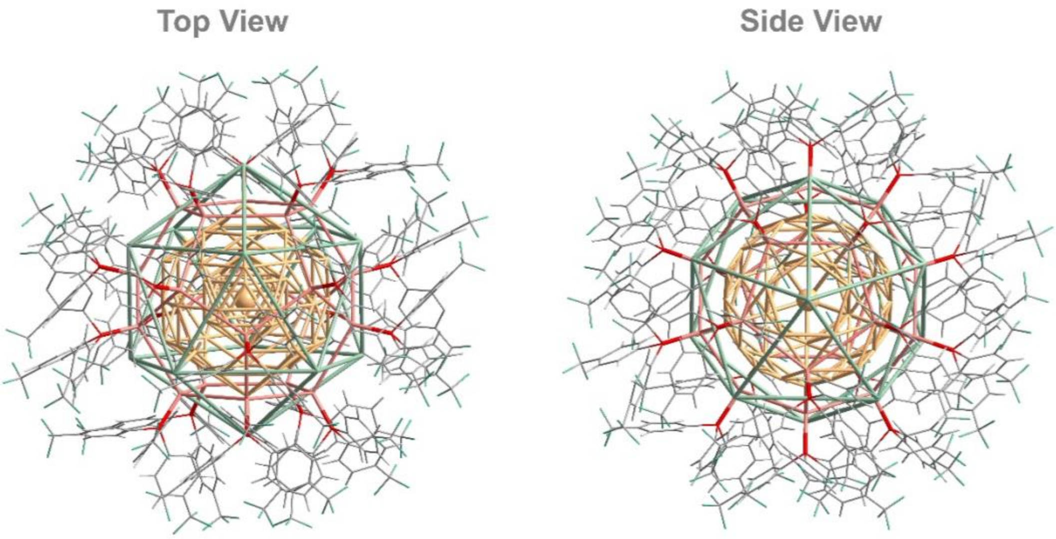

Figure S11. Molecular structure of the Au_{75} nanocluster in top and side views. Color legends: yellow, Au; light green, Cl; red, P; cyan, F; grey, C; light gray, H.

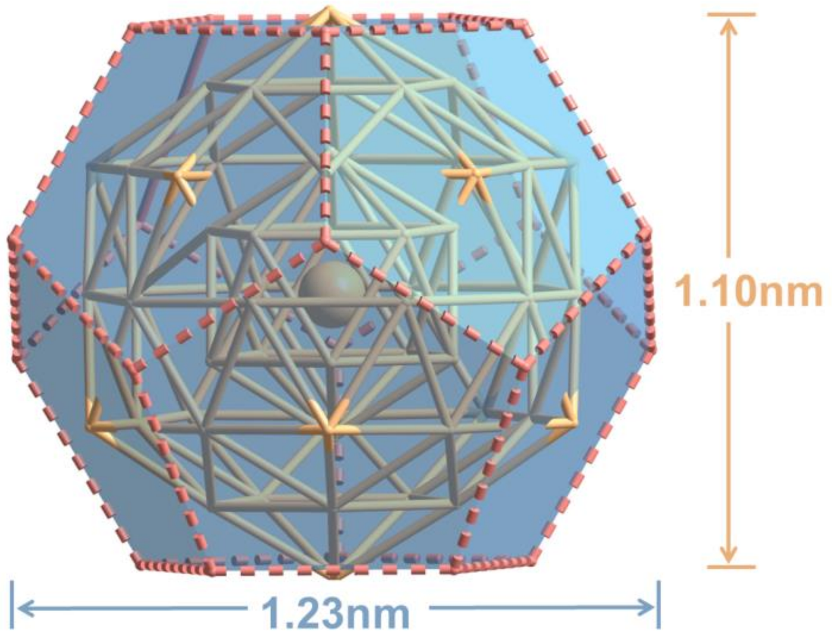

Figure S12. Dimensions of the Au_{55} kernel in the Au_{75} nanocluster. Axial equatorial diameter: 1.23 nm. Axial thickness dimensions: 1.10 nm.

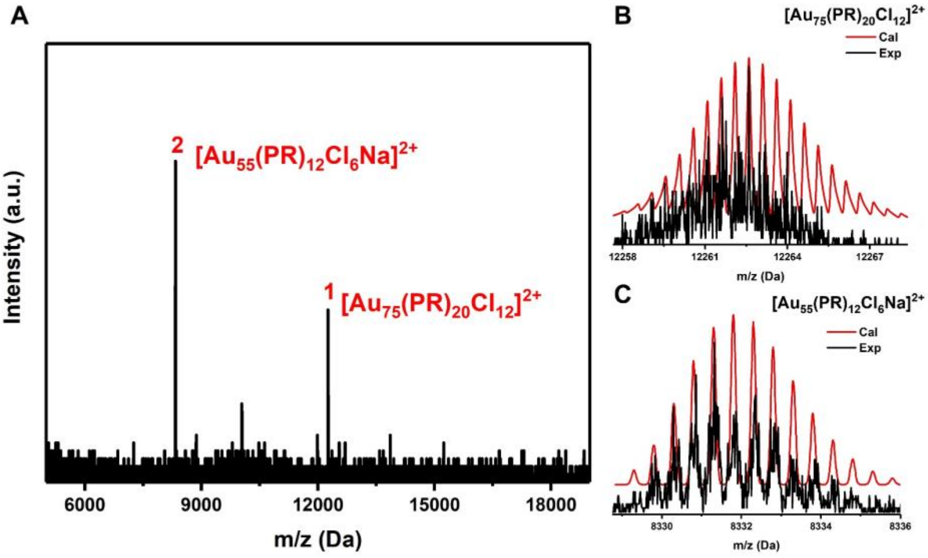

Figure S13. (A) ESI-MS spectrum of the Au_{75} nanocluster, wherein two dominant mass signals were detected. (B) Mass signal of $[Au_{75}(P(C_6H_4\text{-}4\text{-}CF_3)_3)_{20}Cl_{12}]^{2+}$. (C) Mass signal of $[Au_{55}(P(C_6H_4\text{-}4\text{-}CF_3)_3)_{12}Cl_6Na]^{2+}$. Insets: the measured (black trace) and simulated (red trace) isotopic patterns. Due to the difficulty in detecting the mass signal of the Au_{75} nanocluster, we increased detection voltage and temperature in the mass spectrometry. Although the Au_{75} signal was observed under such conditions, its isotope peaks were also unclear; besides, the Au_{55} signal was also detected as a fragment of the Au_{75} nanocluster.

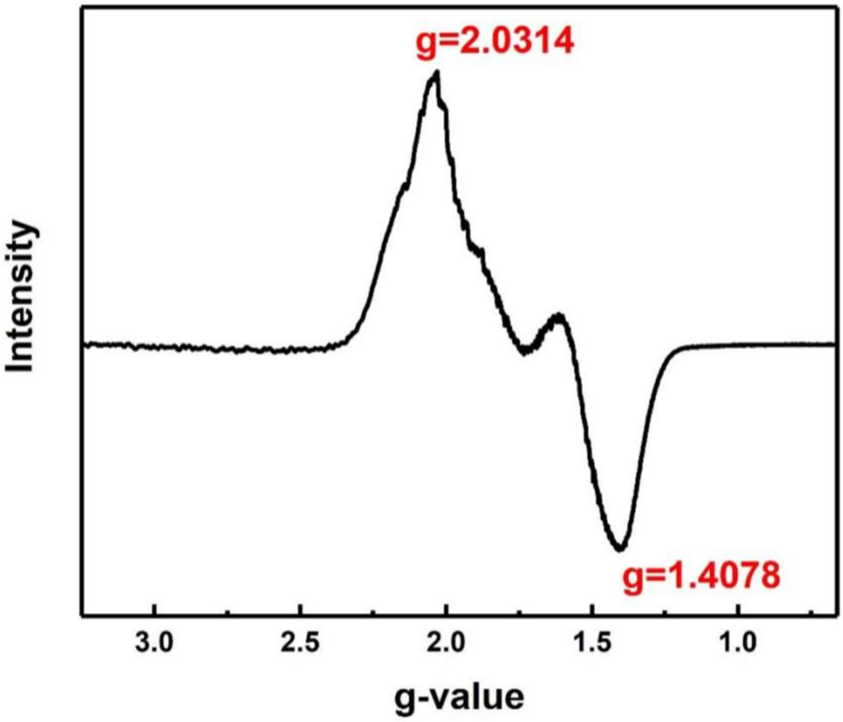

Figure S14. EPR signals of the Au_{75} nanocluster at 2K.

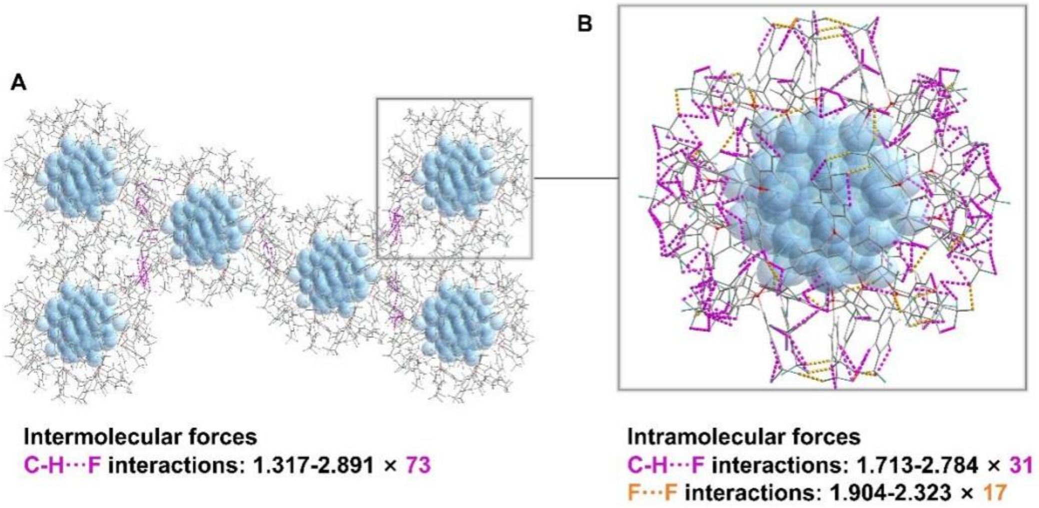

Figure S15. (A) Intercluster C-H···F interactions between adjacent Au_{75} nanoclusters at the supramolecular level. (B) Intracluster C-H···F interactions and F···F interactions of the Au_{75} nanocluster at the molecular level.

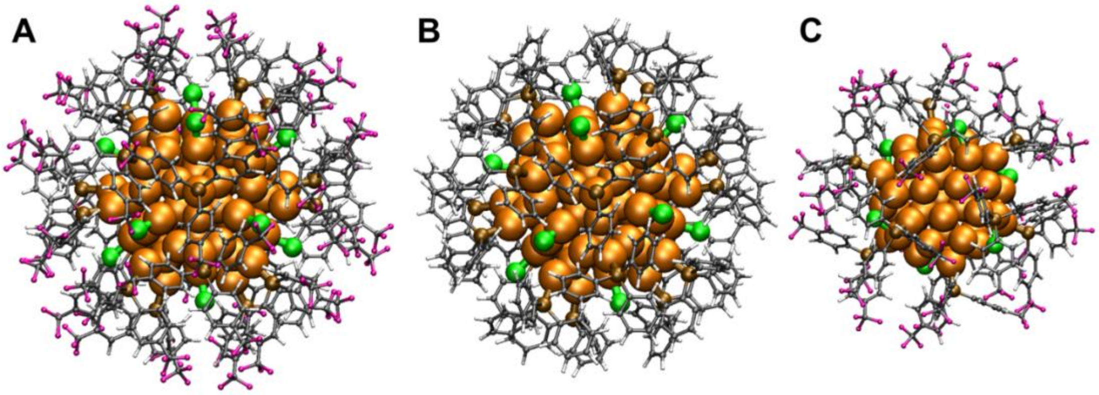

Figure S16. Visualization of (A) $Au_{75}(P(C_6H_4\text{-}4\text{-}CF_3)_3)_{20}Cl_{12}^{2+}$, (B) $Au_{75}(PPh_3)_{20}Cl_{12}^{2+}$, and (C) hypothetical fluorinated "Schmid cluster" $Au_{55}(P(C_6H_4\text{-}4\text{-}CF_3)_3)_{12}Cl_6^{+}$. Au is shown by orange color, Cl green, P brown, C grey, F purple and H white.

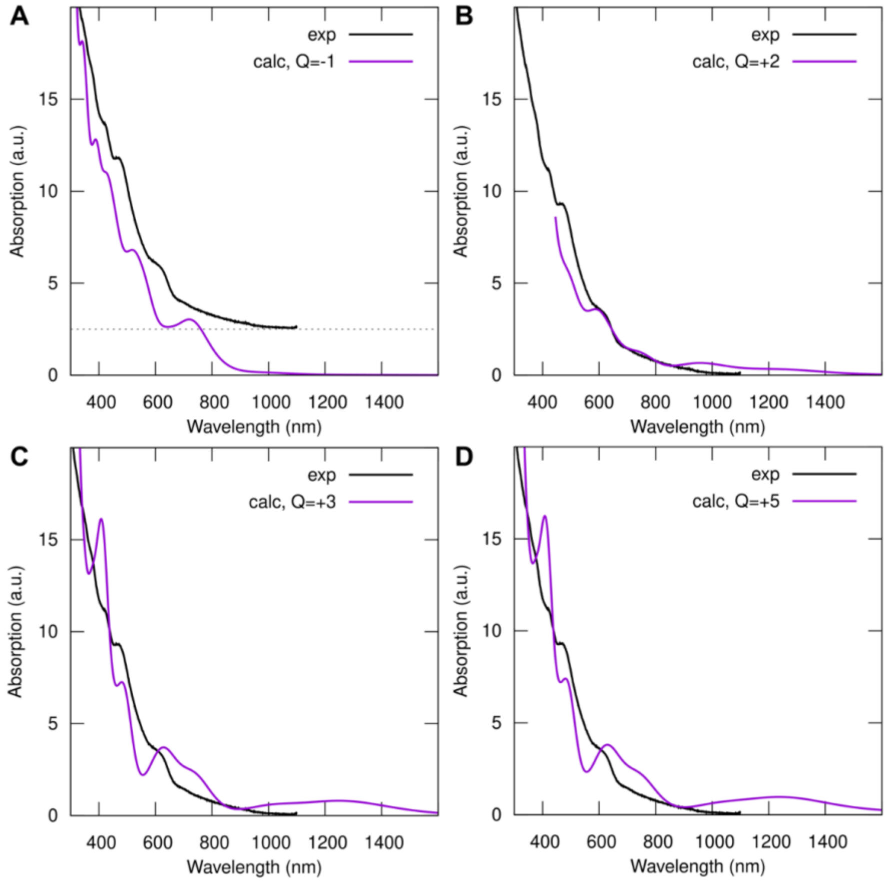

Figure S17. Computed UV-vis spectra of the $Au_{75}(P(C_6H_4\text{-}4\text{-}CF_3)_3)_{20}Cl_{12}^{Q}$ cluster with charges Q shown in the figure panels, as compared to the experimental data.

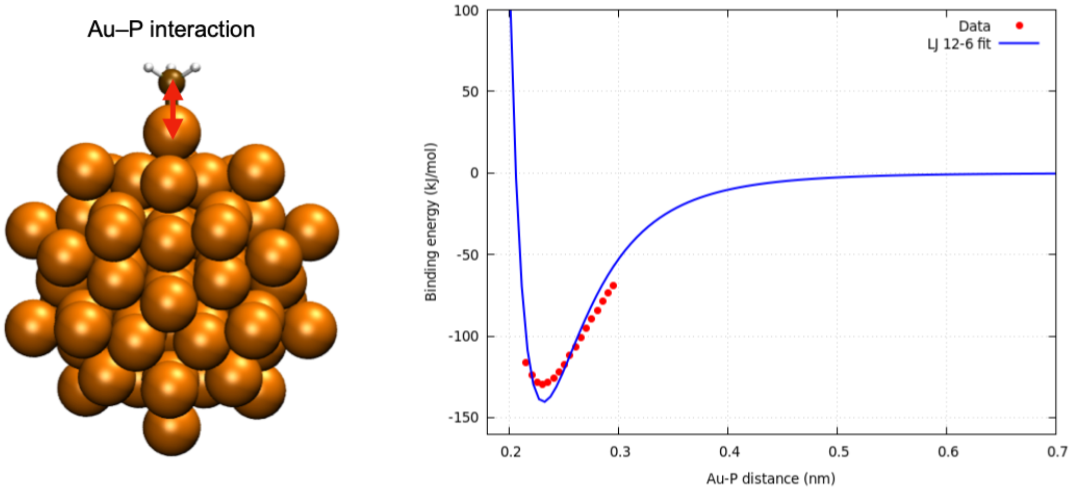

Figure S18. Lennard-Jones potential fitting using $Au_{75}(PH_3)_{20}Cl_{12}^{2+}$ cluster to obtain the non-bonded parameters for Au-P interactions (ε = 140.412 kJ/mol and σ = 0.206 nm). Au is shown by orange color, P brown, and H white. Only the Au_{75} core and one PH_3 ligand are shown for clarity purposes.

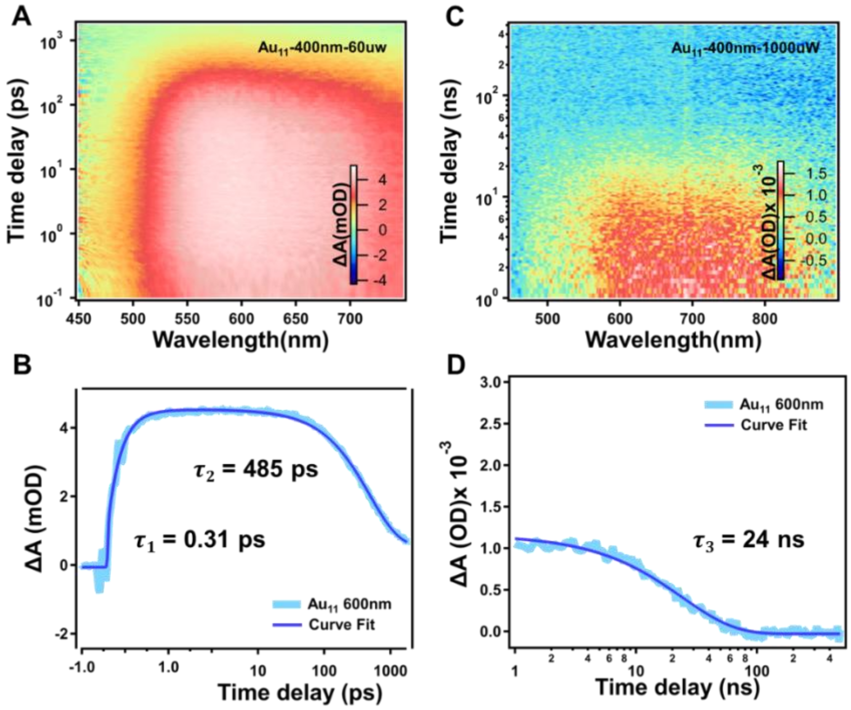

Figure S19. Femtosecond (A) and nanosecond (C) TA data maps of Au_{11} under 400 nm excitation. (B) Femtosecond kinetic trace at 600 nm with dual-exponential fitting. (D) Nanosecond kinetic trace at 600 nm with fitting curve.

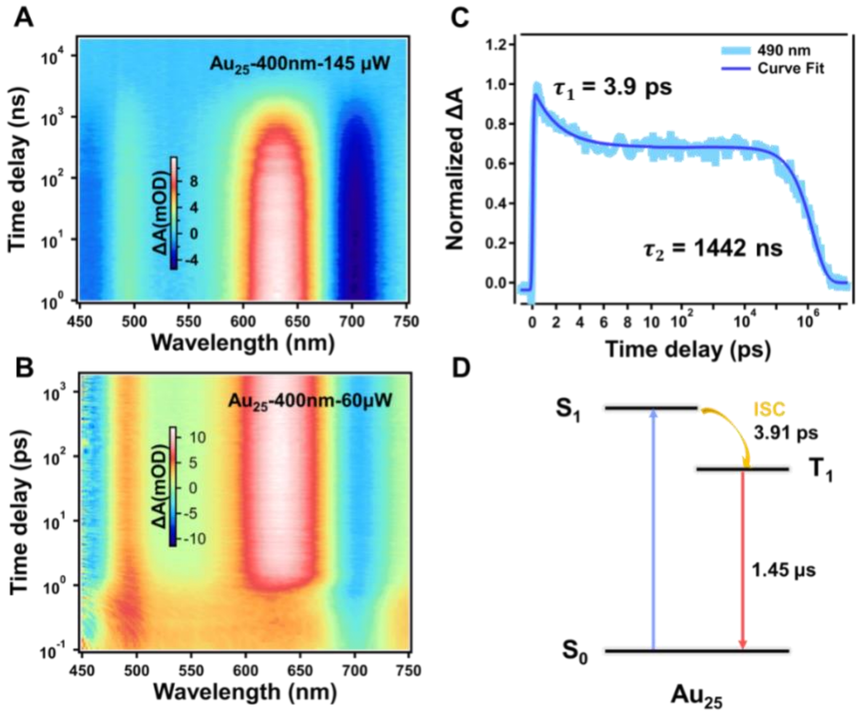

Figure S20. Femtosecond (A) and nanosecond (B) TA data maps of Au_{25} under 400 nm excitation. (C) Merged kinetic trace at 490 nm with dual-exponential fitting. (D) Schematic diagram of excited - state relaxation pathways.

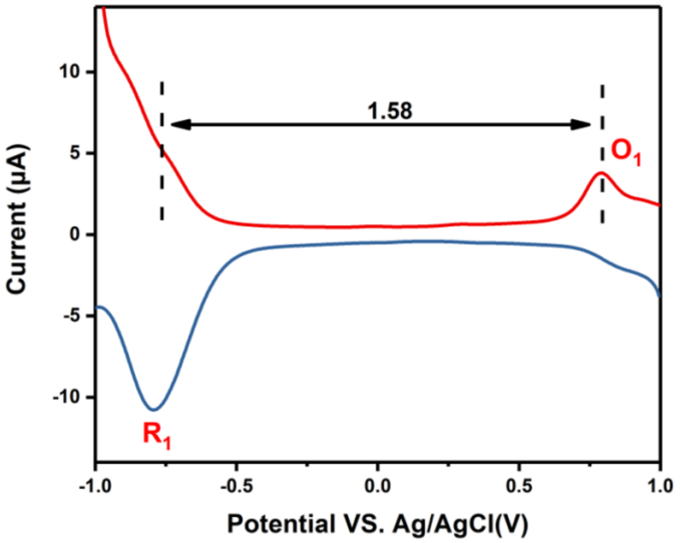

Figure S21. Differential pulse voltammetry results of the Au_{75} nanocluster determine its HOMO-LUMO gap as 1.58 eV.

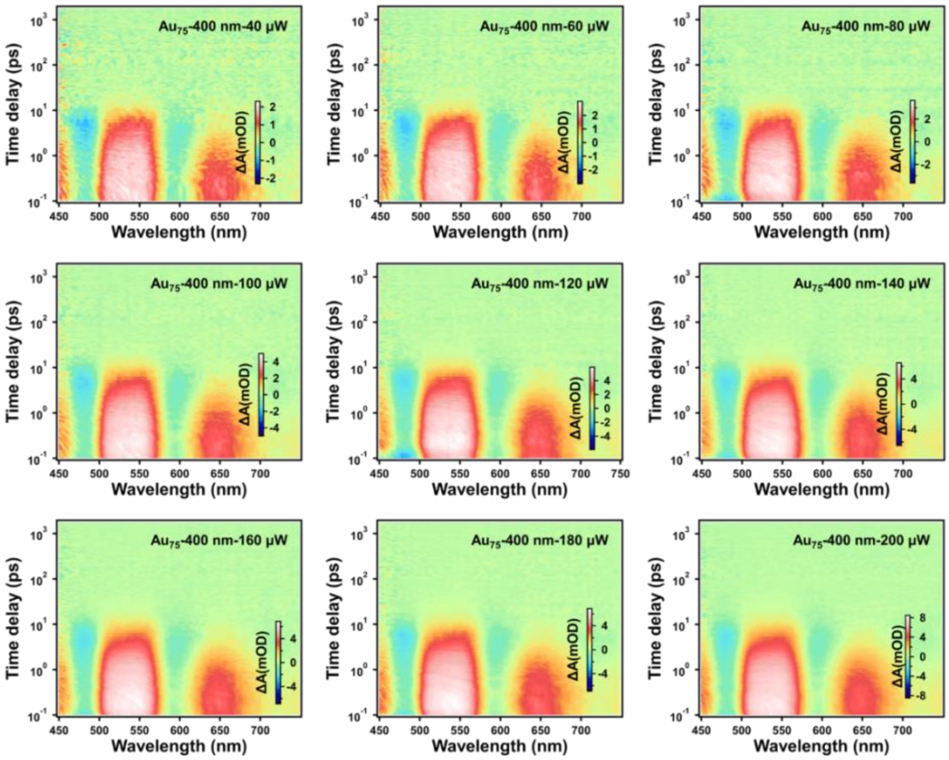

Figure S22. TA data map fs-TA data for Au_{75} nanoclusters under 400 nm excitation, with pump powers ranging from 40 μW to 200 μW.

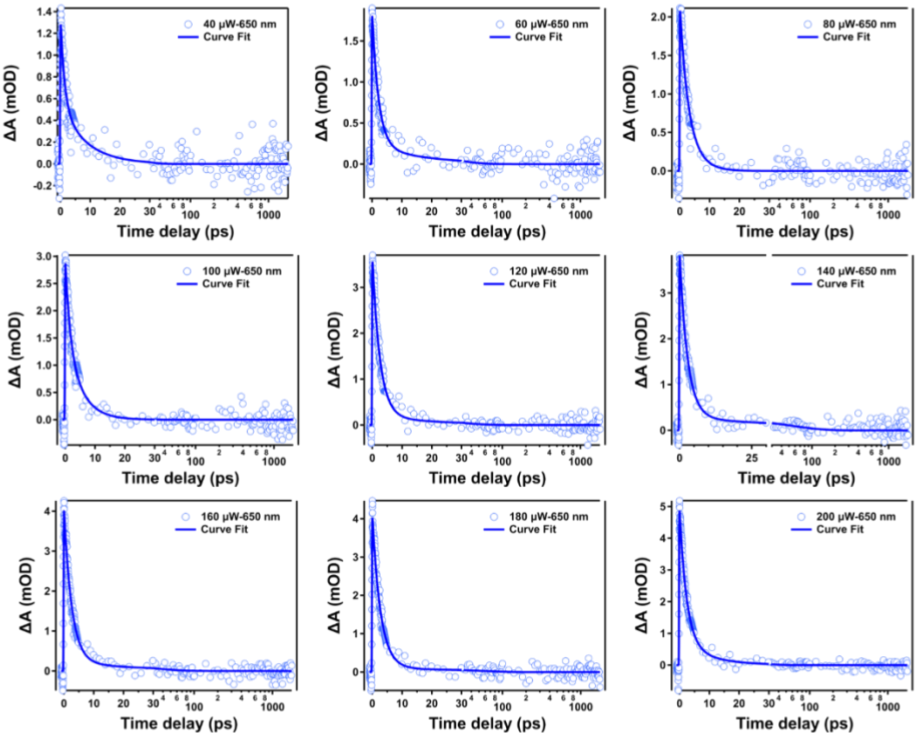

Figure S23. Femtosecond transient absorption kinetic traces at 650 nm for Au_{75}, acquired under 400 nm excitation with pump powers spanning 40–200 μW, together with their corresponding curve fits.

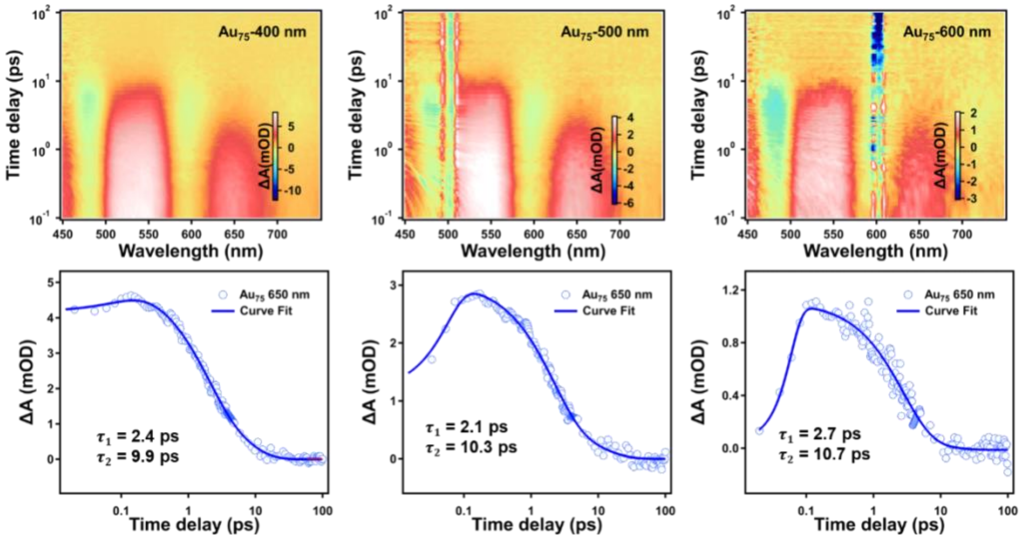

Figure S24. Femtosecond transient absorption data map of Au_{75} nanoclusters under 400 nm (A), 500 nm (C), and 600 nm (E) excitation. (B, D, F) Corresponding kinetic traces at 650 nm (open circles) and their dual-exponential curve fits (solid blue lines), with extracted lifetimes (τ_1, τ_2) labeled.

Table S1. Crystal data and structure refinement for the Au_{75} nanocluster.

Molecular formula	$C_{420}H_{240}F_{180}P_{20}Cl_{14}Au_{75}$
Crystal system	trigonal
Space group	*R*-3
a/Å	28.493(3)
b/Å	28.493(3)
c/Å	64.125(3)
α/°	90
β/°	90
γ/°	120
Volume/Å^3	45086(10)
Z	3
ρ_{calc}g/cm^3	2.717
μ/mm^{-1}	35.333
F (000)	32529.0
Radiation	Cu Kα (λ = 1.54186)
Index ranges	-34 ≤ h ≤ 31, -34 ≤ k ≤ 27, -59 ≤ l ≤ 77
2θ range (°)	6.576 to 139.21
Measured reflections and unique reflections	18679 [R_{int} = 0.0071, R_{sigma} = 0.0249]
Goodness-of-fit on F^2	1.045
Largest diff. peak/hole / e Å^{-3}	2.25/-1.10
Final R indexes [I>=2σ (I)]	R_1 = 0.0319, wR_2 = 0.1026
Final R indexes [all data]	R_1 = 0.0408, wR_2 = 0.1052

Table S2. Crystal data and structure refinement for the Au_{11} nanocluster.

Molecular formula	$C_{147}H_{84}Au_{11}Cl_3F_{61.3}P_7$
Crystal system	triclinic
Space group	*P-1*
a/Å	19.011
b/Å	19.639
c/Å	24.041
α/°	85.19
β/°	87.80
γ/°	81.64
Volume/Å^3	8846.7
Z	2
ρ_{calc}g/cm^3	2.066
μ/mm^{-1}	18.664
F (000)	5085.0
Radiation	Cu Kα (λ = 1.54186)
Index ranges	-15 ≤ h ≤ 23, -23 ≤ k ≤ 23, -29 ≤ l ≤ 22
2θ range (°)	9.408 to 138.99
Measured reflections and unique reflections	63799 [R_{int} = 0.0309, R_{sigma} = 0.0306]
Goodness-of-fit on F^2	1.029
Largest diff. peak/hole / e Å^{-3}	5.10/-4.66
Final R indexes [I>=2σ (I)]	R_1 = 0.0616, wR_2 = 0.1658
Final R indexes [all data]	R_1 = 0.0655, wR_2 = 0.1710

Table S3. Crystal data and structure refinement for the Au_{25} nanocluster.

Molecular formula	$C_{209.82}H_{191.82}Au_{25}Cl_2P_{10}S_5$
Crystal system	monoclinic
Space group	*P2/c*
a/Å	32.685(2)
b/Å	20.8474(12)
c/Å	34.452(3)
α/°	90
β/°	91.123(6)
γ/°	90
Volume/Å^3	23471(3)
Z	4
ρ_{calc}g/cm^3	2.314
μ/mm^{-1}	30.124
F (000)	14759.0
Radiation	Cu Kα (λ = 1.54186)
Index ranges	-28 ≤ h ≤ 39, -25 ≤ k ≤ 16, -41 ≤ l ≤ 40
2θ range (°)	6.658 to 140.464
Measured reflections and unique reflections	196100 [R_{int} = 0.0605, R_{sigma} = 0.0524]
Goodness-of-fit on F^2	1.038
Largest diff. peak/hole / e Å^{-3}	3.61/-3.71
Final R indexes [I>=2σ (I)]	R_1 = 0.0570, wR_2 = 0.1404
Final R indexes [all data]	R_1 = 0.0790, wR_2 = 0.1529

Table S4. A summary of Au-Au bond lengths in the Au_{75} nanocluster.

Bonds	distance/Å	Average value/Å	Average ESD/Å
1st shell: Au-Au bonds in Au_{13}	2.748-2.915	2.791	0.006
2nd shell: Au-Au bonds in Au_{42}	2.875-2.965	2.916	0.006

Table S5. A summary of Au-X bond lengths (X = Au/Cl/P) between different layers in Au_{75}.

Bonds	distance/Å	Average value/Å	Average ESD/Å
3rd shell: Au_{20}-Au_{42}	2.718-2.769	2.739	0.006
4th shell: Cl_{12}-Au_{42}	2.301-2.311	2.306	0.025
5fif shell: PR_{20}-Au_{20}	2.279-2.336	2.311	0.003
